# Supplementary figures and images for: Calling genotypes from public RNA-sequencing data enables identification of genetic variants that affect gene-expression levels
Source: Genome Med. 2015 Mar 27;7(1):30. doi: 10.1186/s13073-015-0152-4 (PMC4423486; doi:10.1186/s13073-015-0152-4)

# Sample exclusion using PCA on gene expression levels

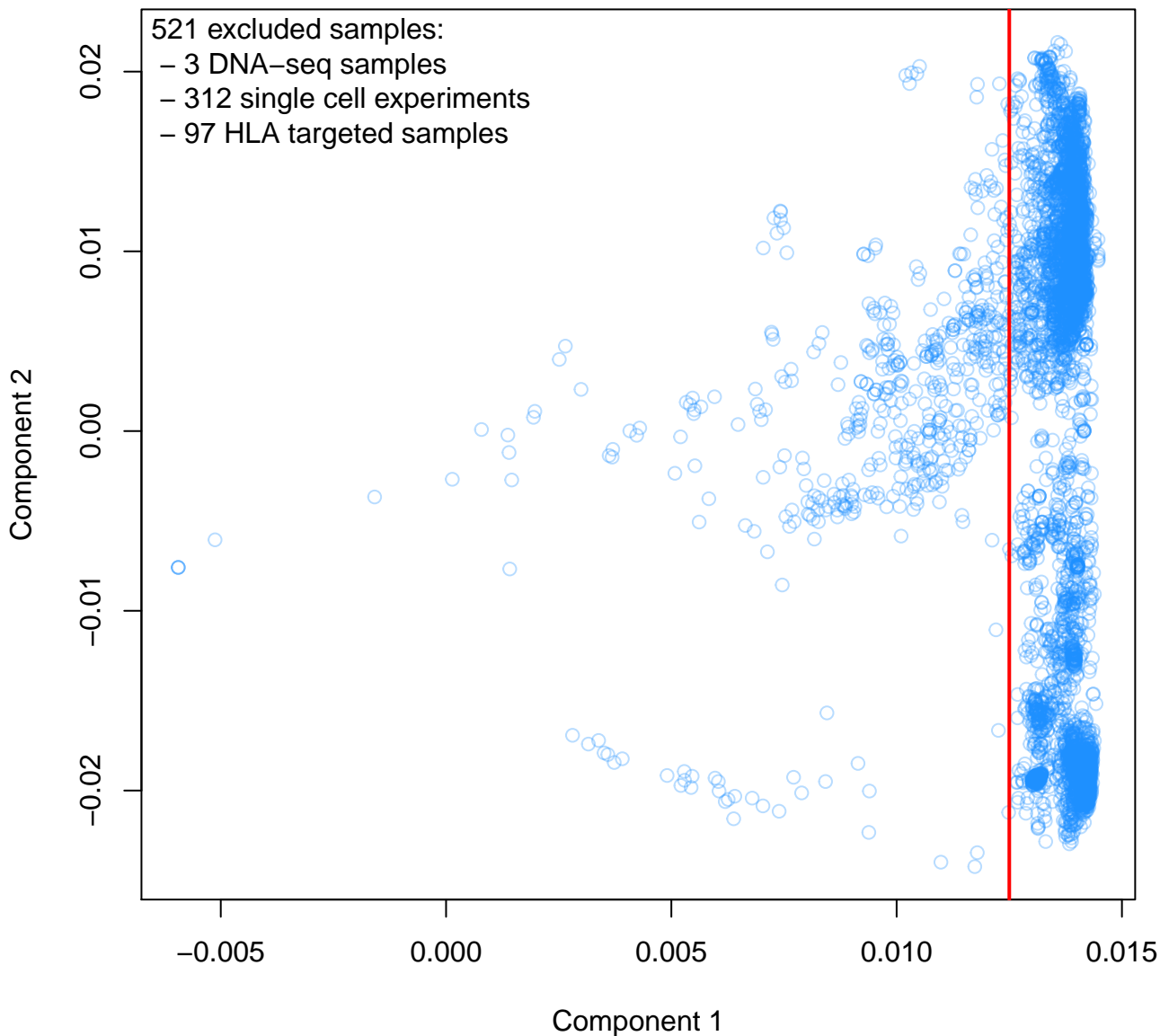

Supplement: Additional file 1: Figure S1. — PCA on expression values shows strong outliers that are removed from the analysis. The 521 outliers of the first component (left of the red line) were removed from our analyses. [file 13073_2015_152_MOESM1_ESM.pdf]

**a**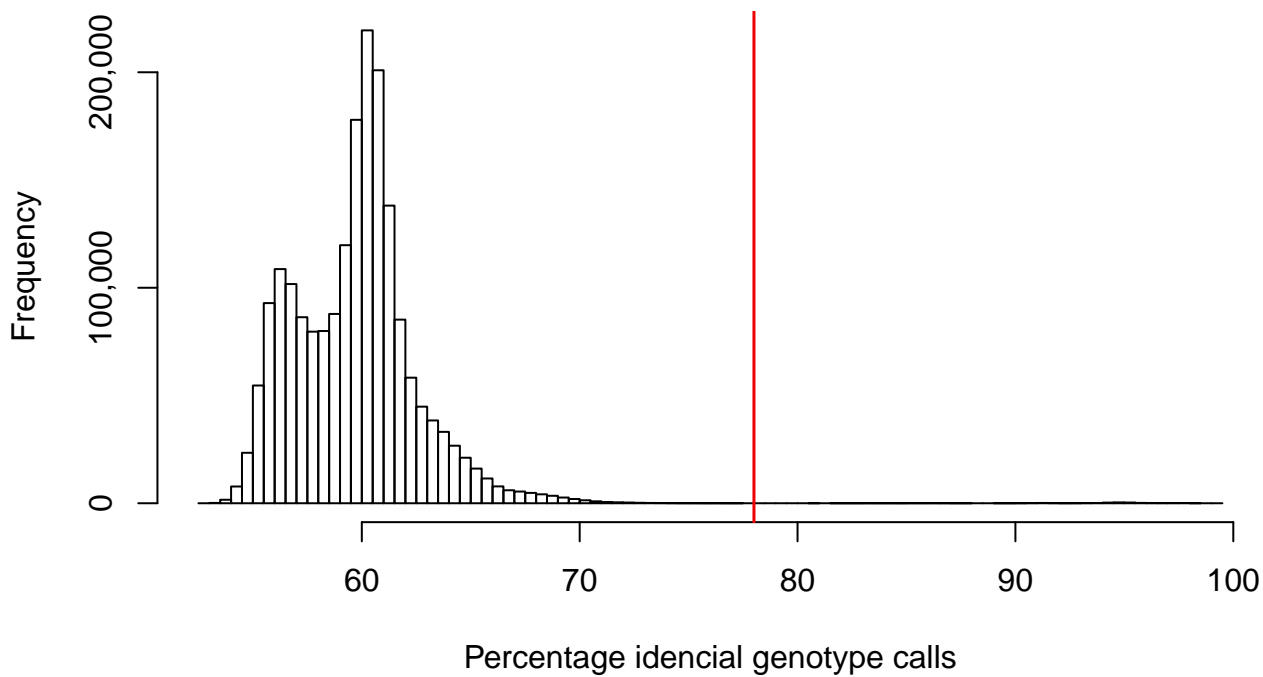**b**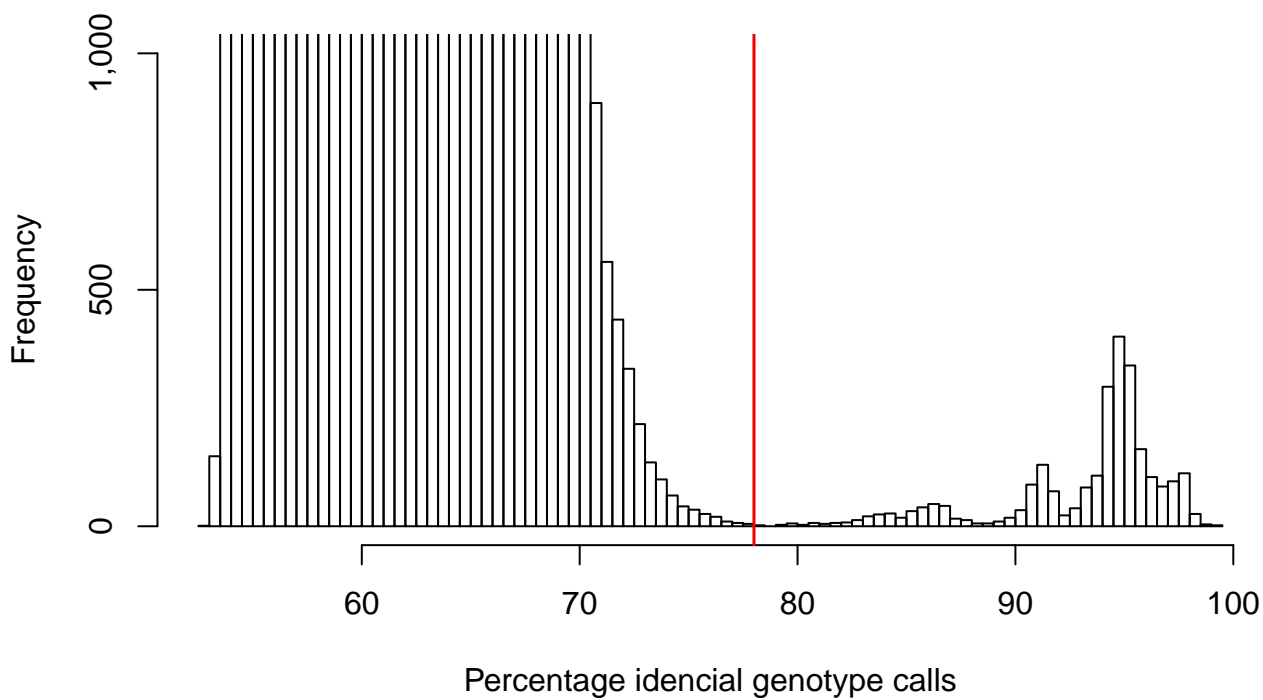

Supplement: Additional file 4: Figure S3. — Identification of duplicate samples. We used a cutoff of 78% identity to select duplicate markers. By using this cutoff level we could identify all the duplicates, which we expected based on the annotations. The reason that we have multiple peaks above this cutoff is due to the difference in genotyping quality among the samples. Panel (b) is the enlargement of the lower part of panel (a). [file 13073_2015_152_MOESM4_ESM.pdf]

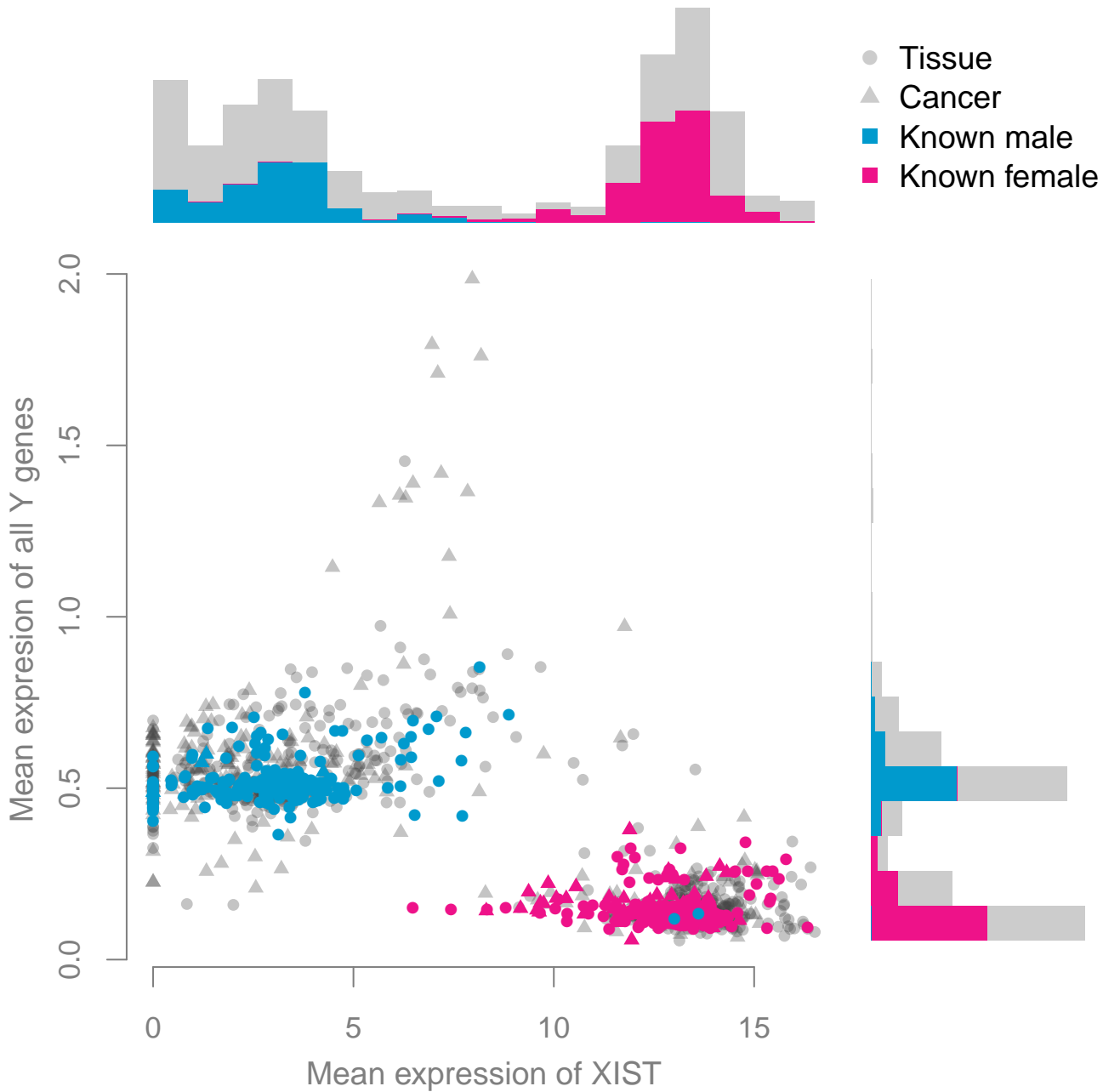

Supplement: Additional file 5: Figure S4. — Expression of XIST and chromosome Y genes. We show a clear separation of males and females using both XIST expression and chromosome Y expression. In two cases, the samples were annotated as male but clustered within the females; these are likely mis-annotations. [file 13073_2015_152_MOESM5_ESM.pdf]

a

**Sequencer model**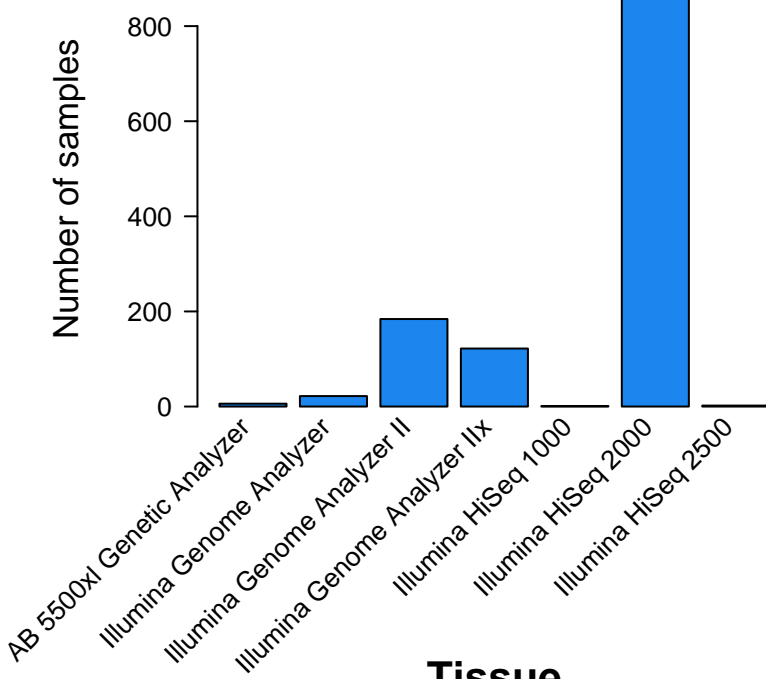

b

**Read layout**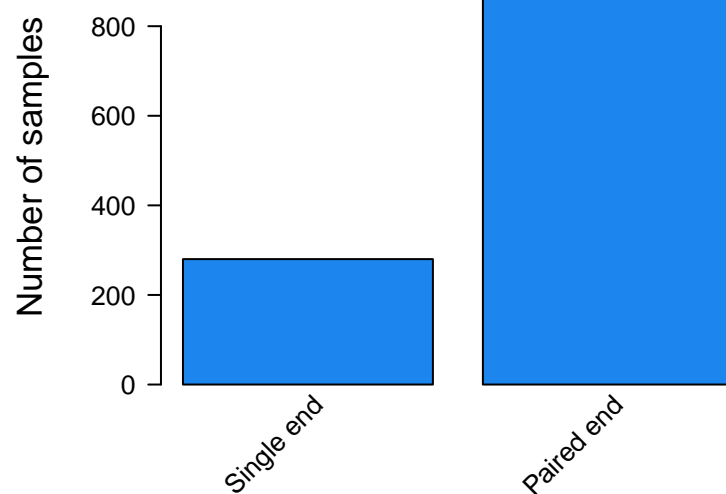

c

**Tissue**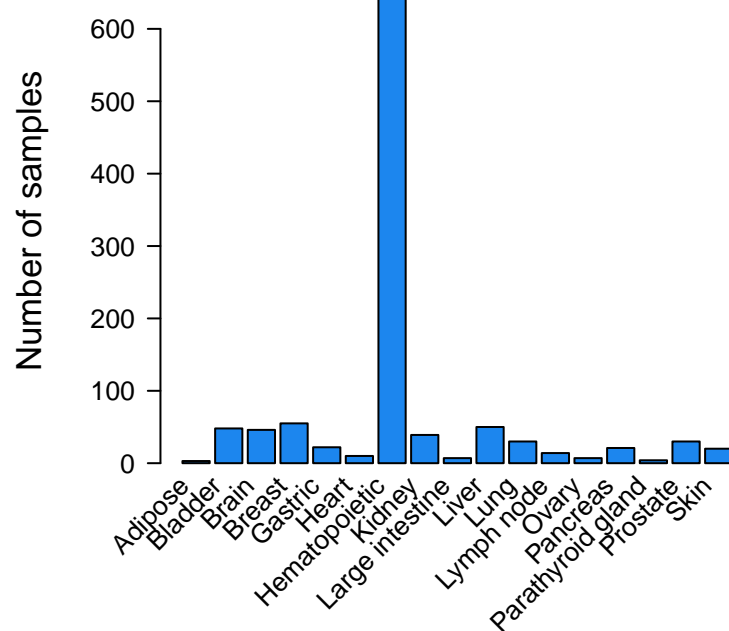

d

**Cancer status**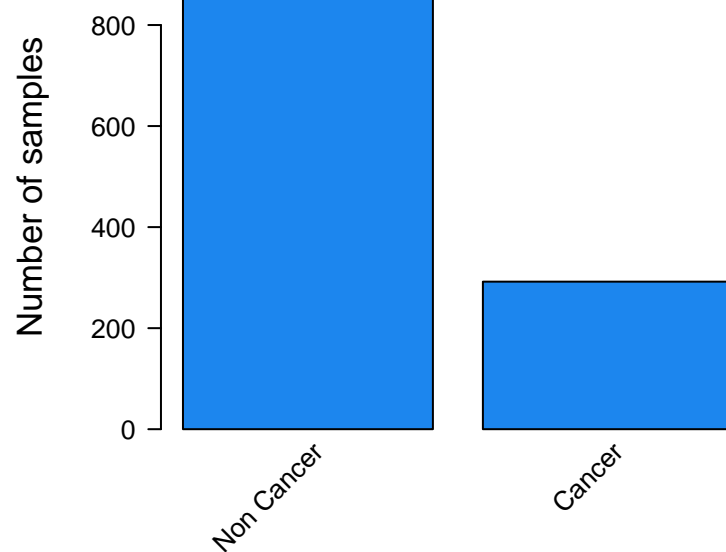

e

**Total Number of reads**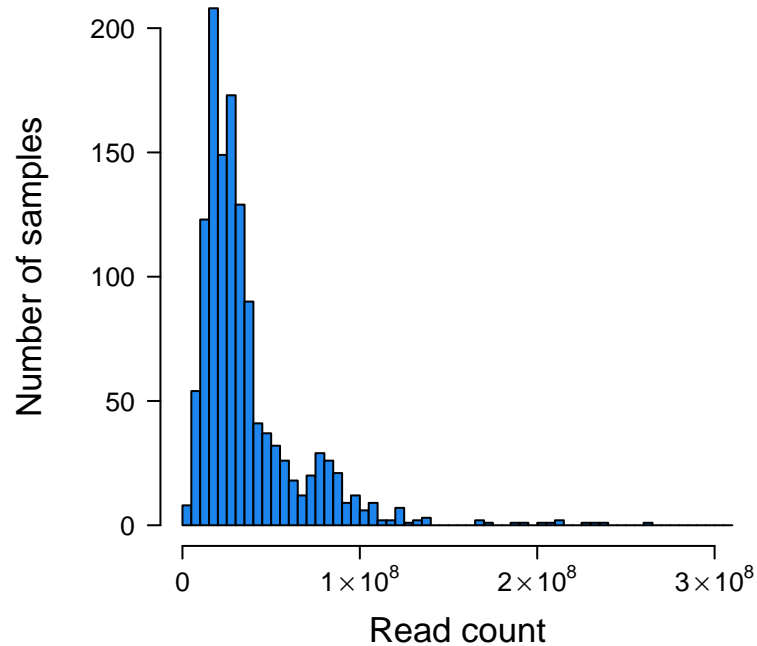

f

**Read length**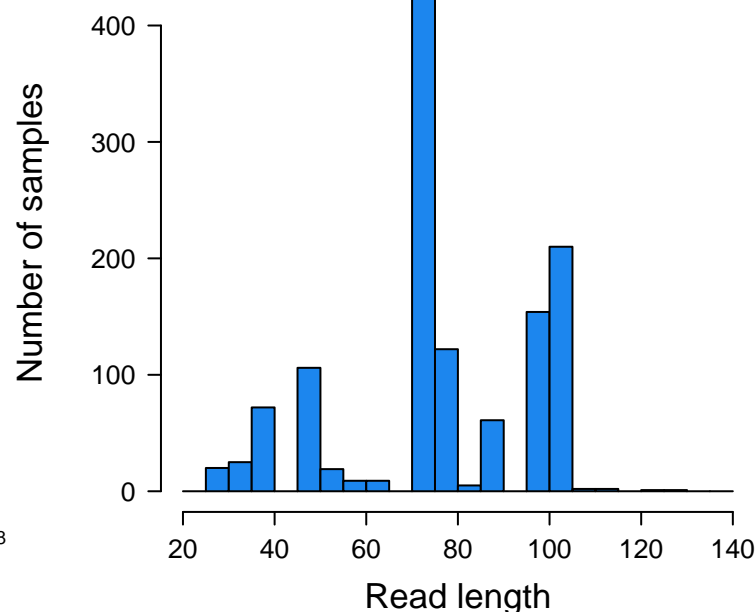

Supplement: Additional file 6: Figure S5. — Overview of the properties of the 1,262 samples used for eQTL and ASE analyses. Here we show that the samples which we successfully genotyped and used for the eQTL and ASE analysis still show high heterogeneity in sequencer models (a), read layout (b), sampled tissue (c), cancer status (d), total number of reads (e), and read length (f). [file 13073_2015_152_MOESM6_ESM.pdf]

**a**

## MASP2 expression in different tissues

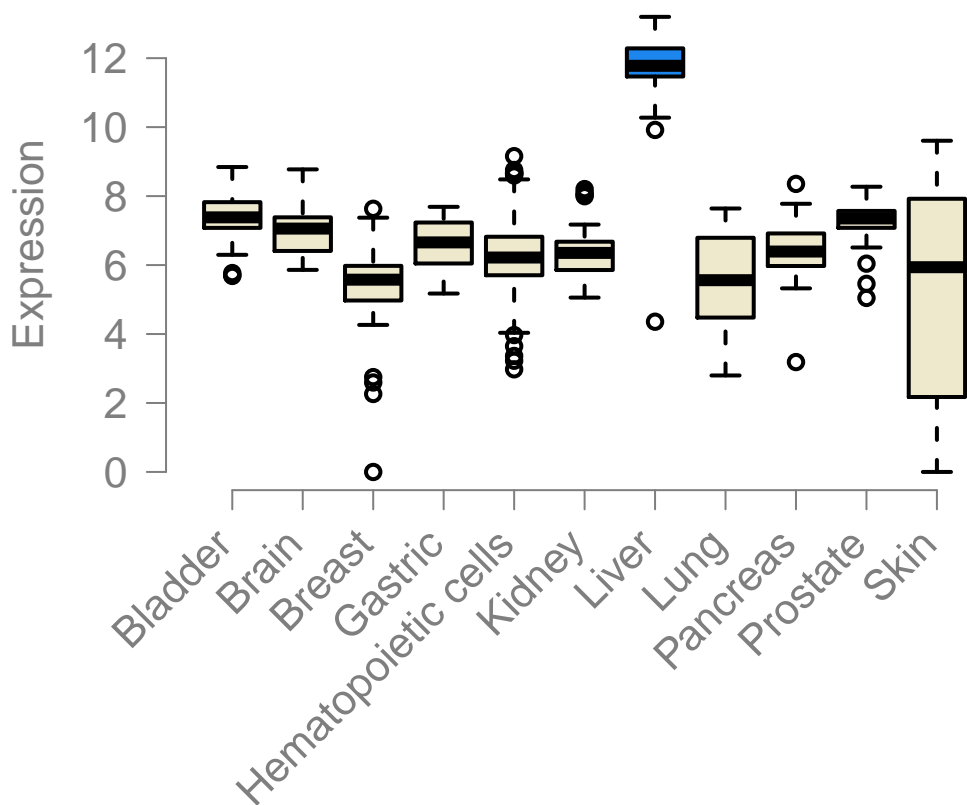**b**

## IRF4 expression in different tissues

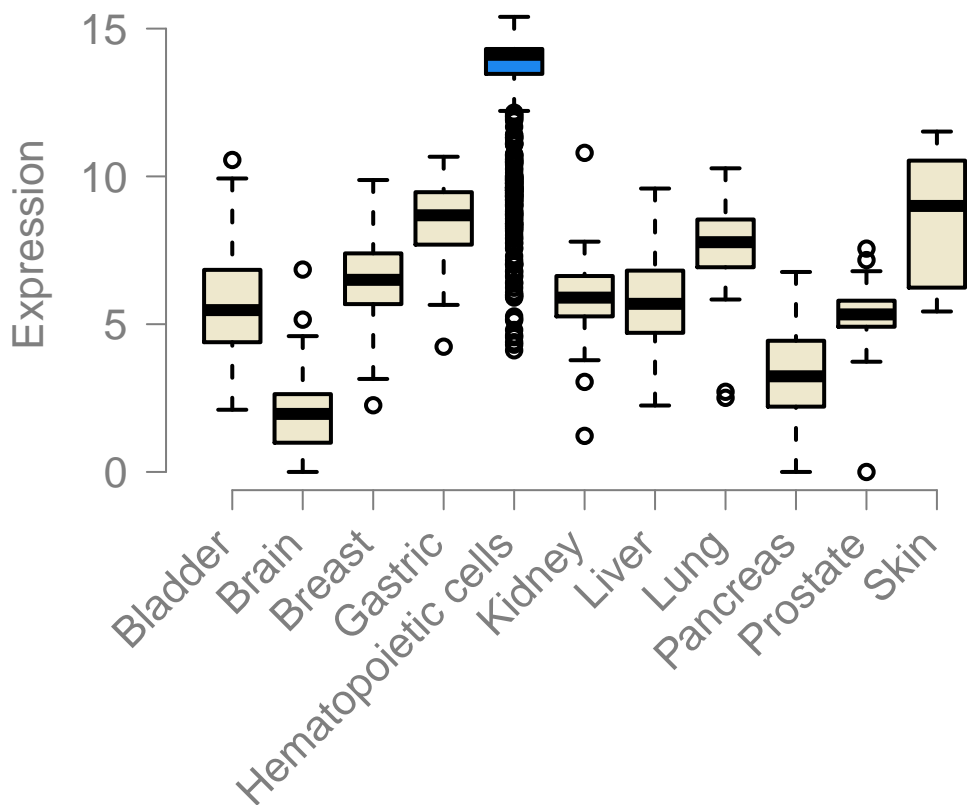

Supplement: Additional file 14: Figure S12. — Expression of the MASP2 gene is liver-specific and the expression of the IRF4 gene is hematopoietic-specific. (a) The MASP2 gene has higher expression in liver compared with other tissues. (b) The IRF4 gene has higher expression in hematopoietic cells compared with other tissues. [file 13073_2015_152_MOESM14_ESM.pdf]
