# Supplementary material for: Calling genotypes from public RNA-sequencing data enables identification of genetic variants that affect gene-expression levels
Source: Genome Med. 2015 Mar 27;7(1):30. doi: 10.1186/s13073-015-0152-4 (PMC4423486; doi:10.1186/s13073-015-0152-4)
Supplement: Additional file 2: Figure S2. — Correlation of principal components versus different confounders. [file 13073_2015_152_MOESM2_ESM.pdf]

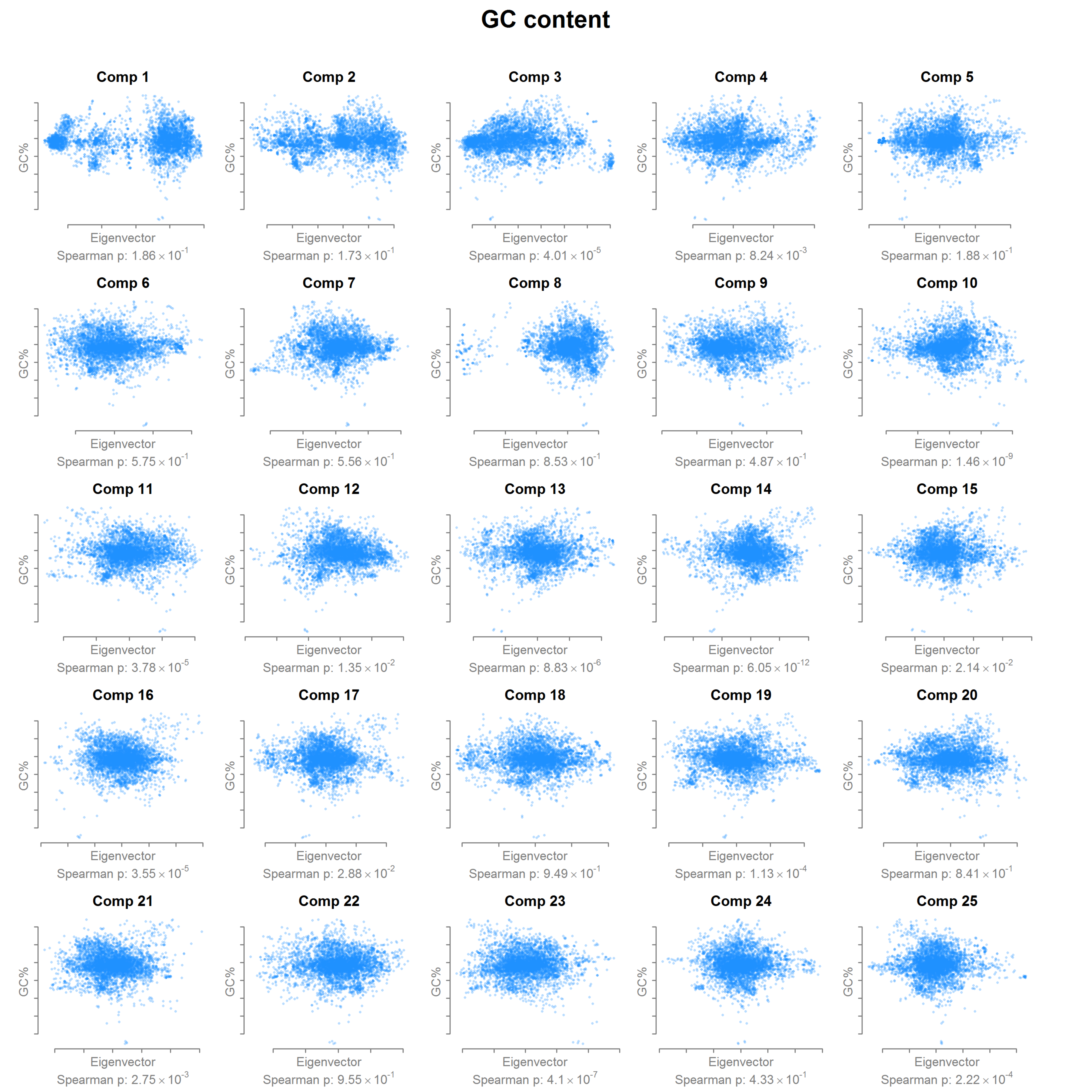
GC content
Comp 1
GC%
Eigenvector
Spearman p: 1.86 × 10^-1
Comp 2
Spearman p: 1.73 × 10^-1
Comp 3
Spearman p: 4.01 × 10^-5
Comp 4
Spearman p: 8.24 × 10^-3
Comp 5
Spearman p: 1.88 × 10^-1
Comp 6
Spearman p: 5.75 × 10^-1
Comp 7
Spearman p: 5.56 × 10^-1
Comp 8
Spearman p: 8.53 × 10^-1
Comp 9
Spearman p: 4.87 × 10^-1
Comp 10
Spearman p: 1.46 × 10^-9
Comp 11
Spearman p: 3.78 × 10^-5
Comp 12
Spearman p: 1.35 × 10^-2
Comp 13
Spearman p: 8.83 × 10^-6
Comp 14
Spearman p: 6.05 × 10^-12
Comp 15
Spearman p: 2.14 × 10^-2
Comp 16
Spearman p: 3.55 × 10^-5
Comp 17
Spearman p: 2.88 × 10^-2
Comp 18
Spearman p: 9.49 × 10^-1
Comp 19
Spearman p: 1.13 × 10^-4
Comp 20
Spearman p: 8.41 × 10^-1
Comp 21
Spearman p: 2.75 × 10^-3
Comp 22
Spearman p: 9.55 × 10^-1
Comp 23
Spearman p: 4.1 × 10^-7
Comp 24
Spearman p: 4.33 × 10^-1
Comp 25
Spearman p: 2.22 × 10^-4

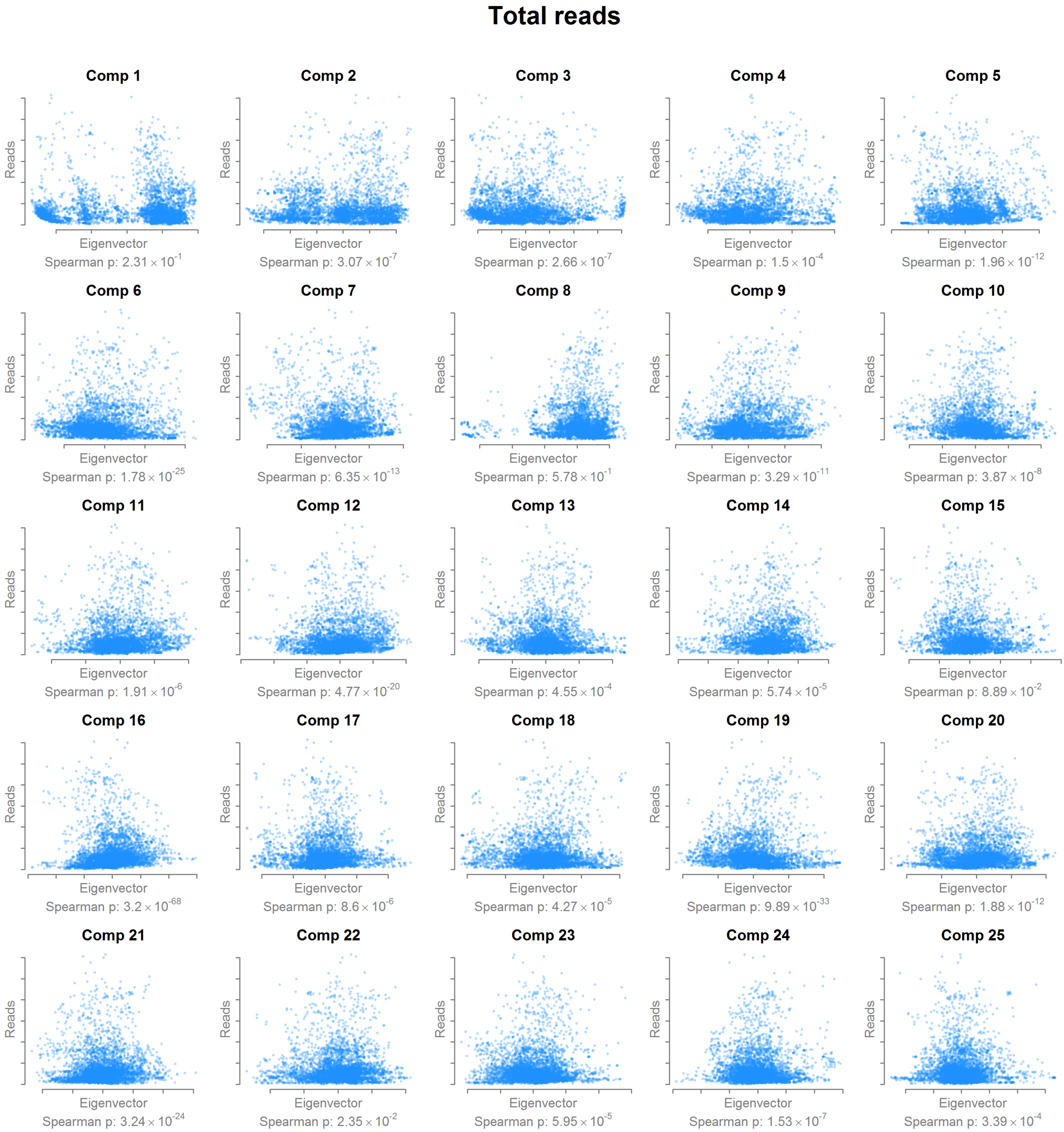
Total reads
Comp 1
Reads
Eigenvector
Spearman p: 2.31×10^{-1}
Comp 2
Reads
Eigenvector
Spearman p: 3.07×10^{-7}
Comp 3
Reads
Eigenvector
Spearman p: 2.66×10^{-7}
Comp 4
Reads
Eigenvector
Spearman p: 1.5×10^{-4}
Comp 5
Reads
Eigenvector
Spearman p: 1.96×10^{-12}
Comp 6
Reads
Eigenvector
Spearman p: 1.78×10^{-25}
Comp 7
Reads
Eigenvector
Spearman p: 6.35×10^{-13}
Comp 8
Reads
Eigenvector
Spearman p: 5.78×10^{-1}
Comp 9
Reads
Eigenvector
Spearman p: 3.29×10^{-11}
Comp 10
Reads
Eigenvector
Spearman p: 3.87×10^{-8}
Comp 11
Reads
Eigenvector
Spearman p: 1.91×10^{-6}
Comp 12
Reads
Eigenvector
Spearman p: 4.77×10^{-20}
Comp 13
Reads
Eigenvector
Spearman p: 4.55×10^{-4}
Comp 14
Reads
Eigenvector
Spearman p: 5.74×10^{-5}
Comp 15
Reads
Eigenvector
Spearman p: 8.89×10^{-2}
Comp 16
Reads
Eigenvector
Spearman p: 3.2×10^{-68}
Comp 17
Reads
Eigenvector
Spearman p: 8.6×10^{-6}
Comp 18
Reads
Eigenvector
Spearman p: 4.27×10^{-5}
Comp 19
Reads
Eigenvector
Spearman p: 9.89×10^{-33}
Comp 20
Reads
Eigenvector
Spearman p: 1.88×10^{-12}
Comp 21
Reads
Eigenvector
Spearman p: 3.24×10^{-24}
Comp 22
Reads
Eigenvector
Spearman p: 2.35×10^{-2}
Comp 23
Reads
Eigenvector
Spearman p: 5.95×10^{-5}
Comp 24
Reads
Eigenvector
Spearman p: 1.53×10^{-7}
Comp 25
Reads
Eigenvector
Spearman p: 3.39×10^{-4}

Uniquely Mapping reads

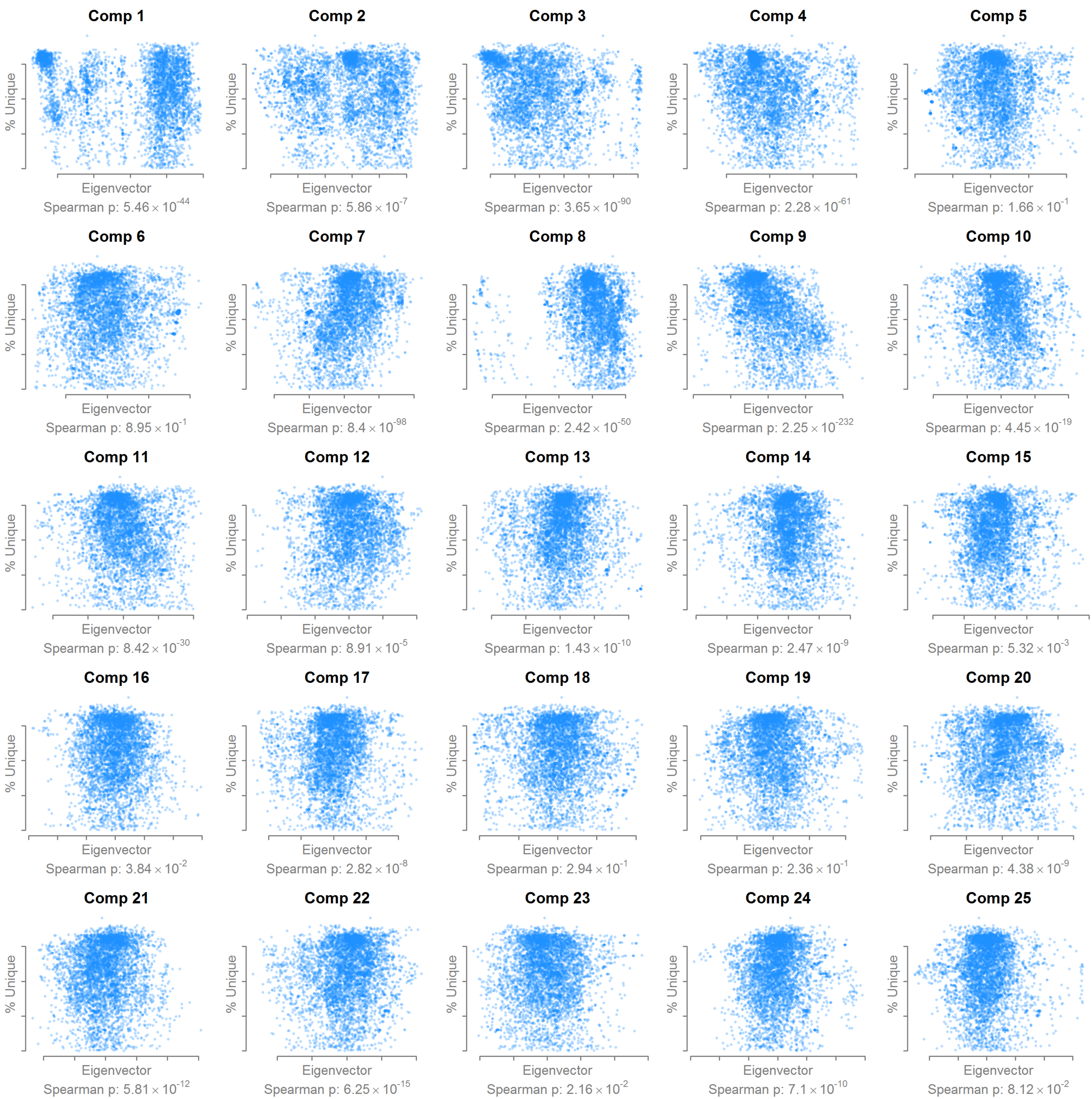

Single (S) vs paired (P) end

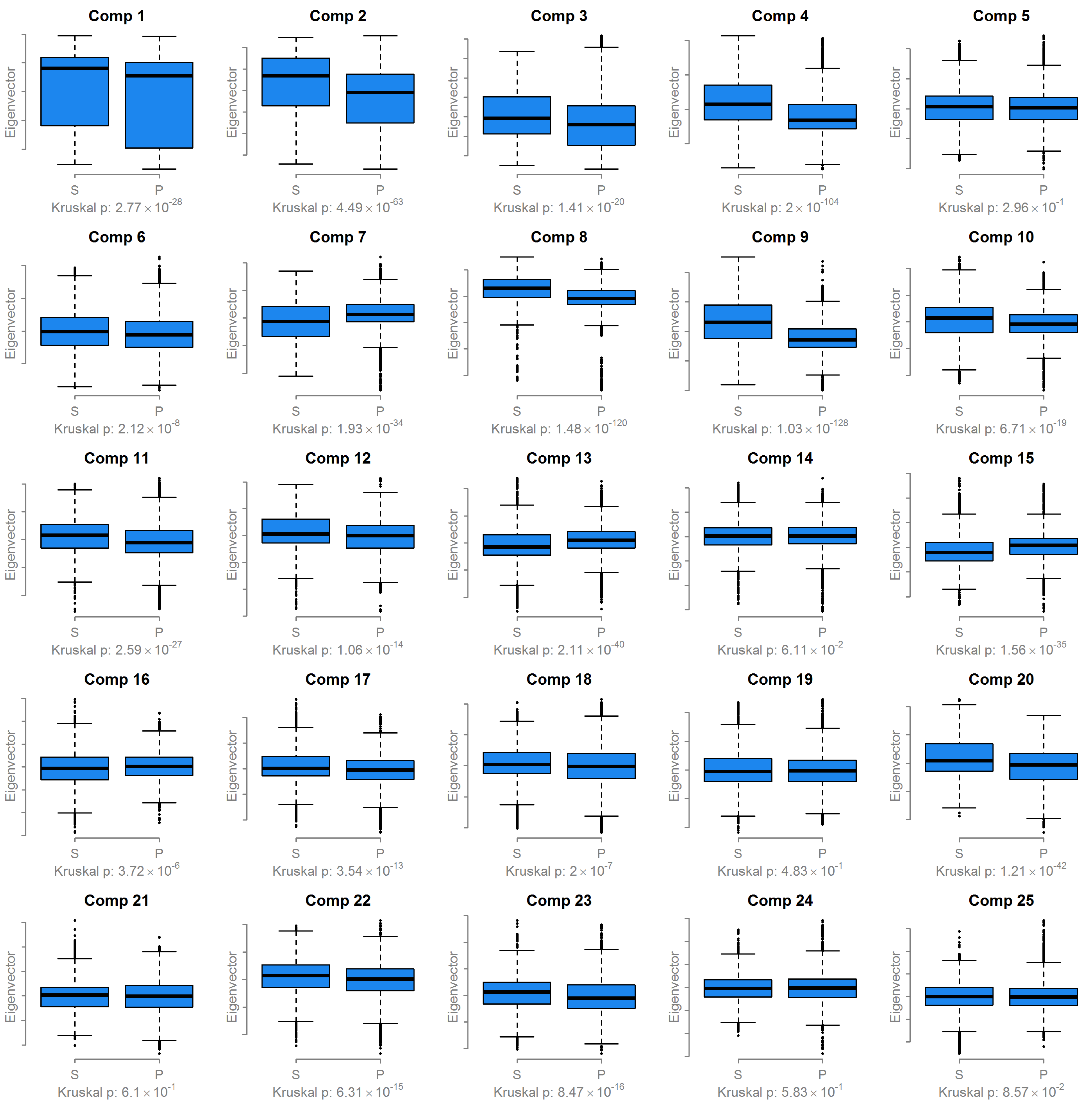

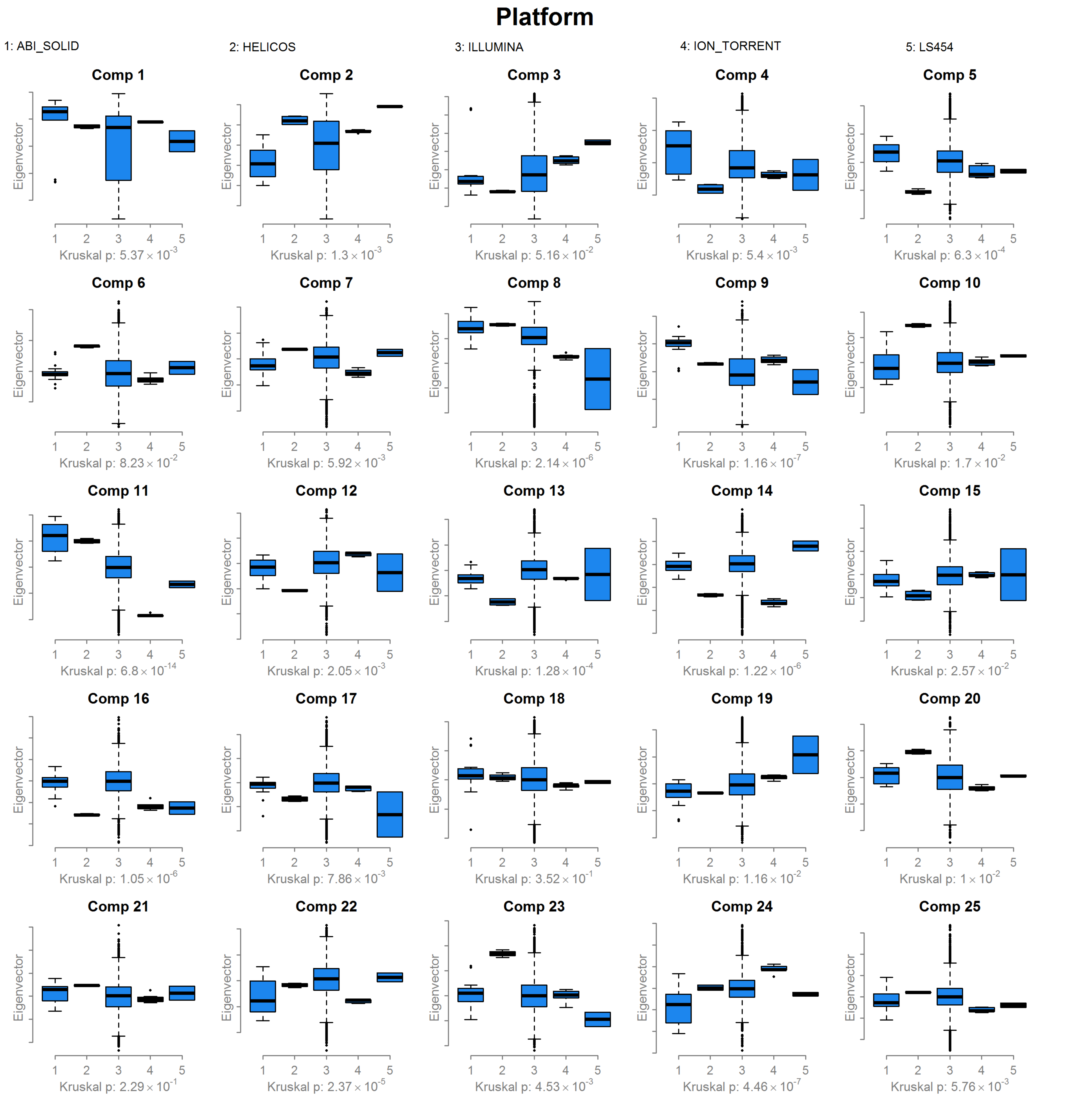
Platform
1: ABI_SOLID
2: HELICOS
3: ILLUMINA
4: ION_TORRENT
5: LS454
Comp 1
Kruskal p: 5.37 × 10^-3
Comp 2
Kruskal p: 1.3 × 10^-3
Comp 3
Kruskal p: 5.16 × 10^-2
Comp 4
Kruskal p: 5.4 × 10^-3
Comp 5
Kruskal p: 6.3 × 10^-4
Comp 6
Kruskal p: 8.23 × 10^-2
Comp 7
Kruskal p: 5.92 × 10^-3
Comp 8
Kruskal p: 2.14 × 10^-6
Comp 9
Kruskal p: 1.16 × 10^-7
Comp 10
Kruskal p: 1.7 × 10^-2
Comp 11
Kruskal p: 6.8 × 10^-14
Comp 12
Kruskal p: 2.05 × 10^-3
Comp 13
Kruskal p: 1.28 × 10^-4
Comp 14
Kruskal p: 1.22 × 10^-6
Comp 15
Kruskal p: 2.57 × 10^-2
Comp 16
Kruskal p: 1.05 × 10^-6
Comp 17
Kruskal p: 7.86 × 10^-3
Comp 18
Kruskal p: 3.52 × 10^-1
Comp 19
Kruskal p: 1.16 × 10^-2
Comp 20
Kruskal p: 1 × 10^-2
Comp 21
Kruskal p: 2.29 × 10^-1
Comp 22
Kruskal p: 2.37 × 10^-5
Comp 23
Kruskal p: 4.53 × 10^-3
Comp 24
Kruskal p: 4.46 × 10^-7
Comp 25
Kruskal p: 5.76 × 10^-3
Eigenvector
1 2 3 4 5

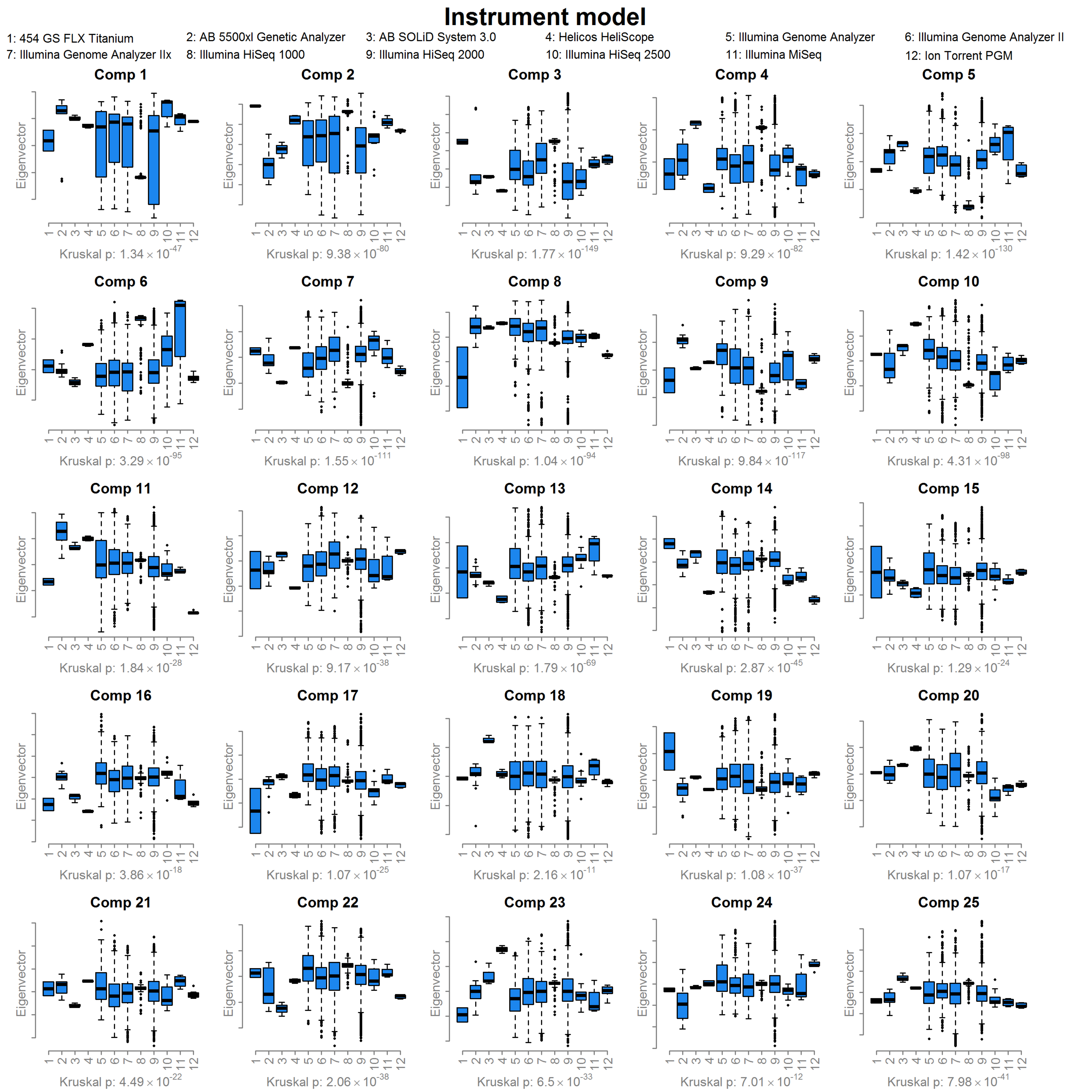

Instrument model
1: 454 GS FLX Titanium
2: AB 5500xl Genetic Analyzer
3: AB SOLiD System 3.0
4: Helicos HeliScope
5: Illumina Genome Analyzer
6: Illumina Genome Analyzer II
7: Illumina Genome Analyzer IIx
8: Illumina HiSeq 1000
9: Illumina HiSeq 2000
10: Illumina HiSeq 2500
11: Illumina MiSeq
12: Ion Torrent PGM
Eigenvector
Comp 1
Kruskal p: 1.34×10^{-47}
Comp 2
Kruskal p: 9.38×10^{-80}
Comp 3
Kruskal p: 1.77×10^{-149}
Comp 4
Kruskal p: 9.29×10^{-82}
Comp 5
Kruskal p: 1.42×10^{-130}
Comp 6
Kruskal p: 3.29×10^{-95}
Comp 7
Kruskal p: 1.55×10^{-111}
Comp 8
Kruskal p: 1.04×10^{-94}
Comp 9
Kruskal p: 9.84×10^{-117}
Comp 10
Kruskal p: 4.31×10^{-98}
Comp 11
Kruskal p: 1.84×10^{-28}
Comp 12
Kruskal p: 9.17×10^{-38}
Comp 13
Kruskal p: 1.79×10^{-69}
Comp 14
Kruskal p: 2.87×10^{-45}
Comp 15
Kruskal p: 1.29×10^{-24}
Comp 16
Kruskal p: 3.86×10^{-18}
Comp 17
Kruskal p: 1.07×10^{-25}
Comp 18
Kruskal p: 2.16×10^{-11}
Comp 19
Kruskal p: 1.08×10^{-37}
Comp 20
Kruskal p: 1.07×10^{-17}
Comp 21
Kruskal p: 4.49×10^{-22}
Comp 22
Kruskal p: 2.06×10^{-38}
Comp 23
Kruskal p: 6.5×10^{-33}
Comp 24
Kruskal p: 7.01×10^{-12}
Comp 25
Kruskal p: 7.98×10^{-41}

Gender

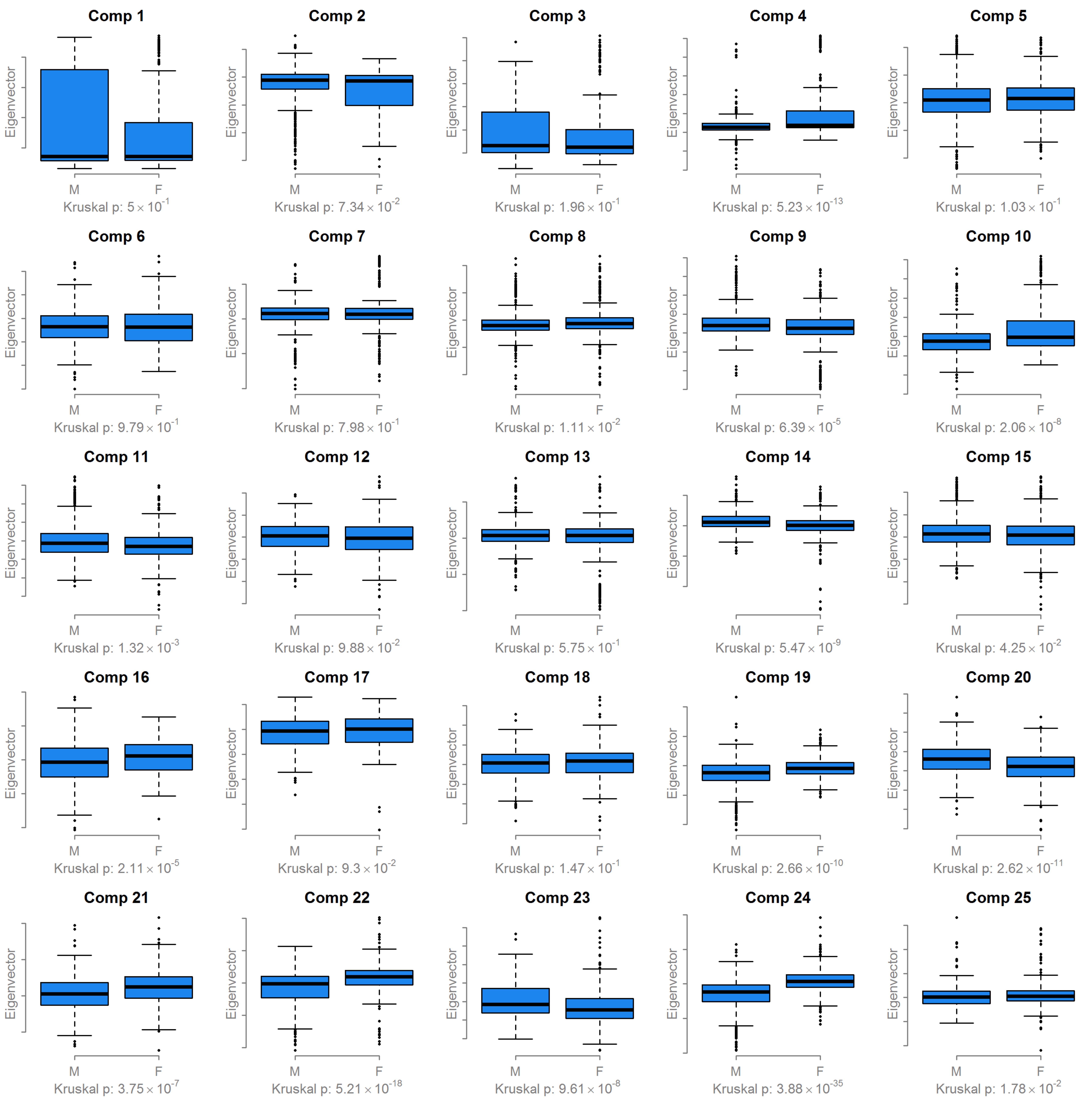
Comp 1
Eigenvector
M
F
Kruskal p: 5×10^{-1}
Comp 2
Kruskal p: 7.34×10^{-2}
Comp 3
Kruskal p: 1.96×10^{-1}
Comp 4
Kruskal p: 5.23×10^{-13}
Comp 5
Kruskal p: 1.03×10^{-1}
Comp 6
Kruskal p: 9.79×10^{-1}
Comp 7
Kruskal p: 7.98×10^{-1}
Comp 8
Kruskal p: 1.11×10^{-2}
Comp 9
Kruskal p: 6.39×10^{-5}
Comp 10
Kruskal p: 2.06×10^{-8}
Comp 11
Kruskal p: 1.32×10^{-3}
Comp 12
Kruskal p: 9.88×10^{-2}
Comp 13
Kruskal p: 5.75×10^{-1}
Comp 14
Kruskal p: 5.47×10^{-9}
Comp 15
Kruskal p: 4.25×10^{-2}
Comp 16
Kruskal p: 2.11×10^{-5}
Comp 17
Kruskal p: 9.3×10^{-2}
Comp 18
Kruskal p: 1.47×10^{-1}
Comp 19
Kruskal p: 2.66×10^{-10}
Comp 20
Kruskal p: 2.62×10^{-11}
Comp 21
Kruskal p: 3.75×10^{-7}
Comp 22
Kruskal p: 5.21×10^{-18}
Comp 23
Kruskal p: 9.61×10^{-8}
Comp 24
Kruskal p: 3.88×10^{-35}
Comp 25
Kruskal p: 1.78×10^{-2}
