# Supplementary material for: Calling genotypes from public RNA-sequencing data enables identification of genetic variants that affect gene-expression levels
Source: Genome Med. 2015 Mar 27;7(1):30. doi: 10.1186/s13073-015-0152-4 (PMC4423486; doi:10.1186/s13073-015-0152-4)
Supplement: Additional file 7: Figure S6. — The relation between sequencing depth and the number of high quality genotypes. (a) We observe a strong relation between the number of sequenced bases and the number of high quality genotypes, we do not observe that paired end sequencing improves genotyping. (b) We observe that newer samples usually have more bases sequenced. [file 13073_2015_152_MOESM7_ESM.pdf]

a

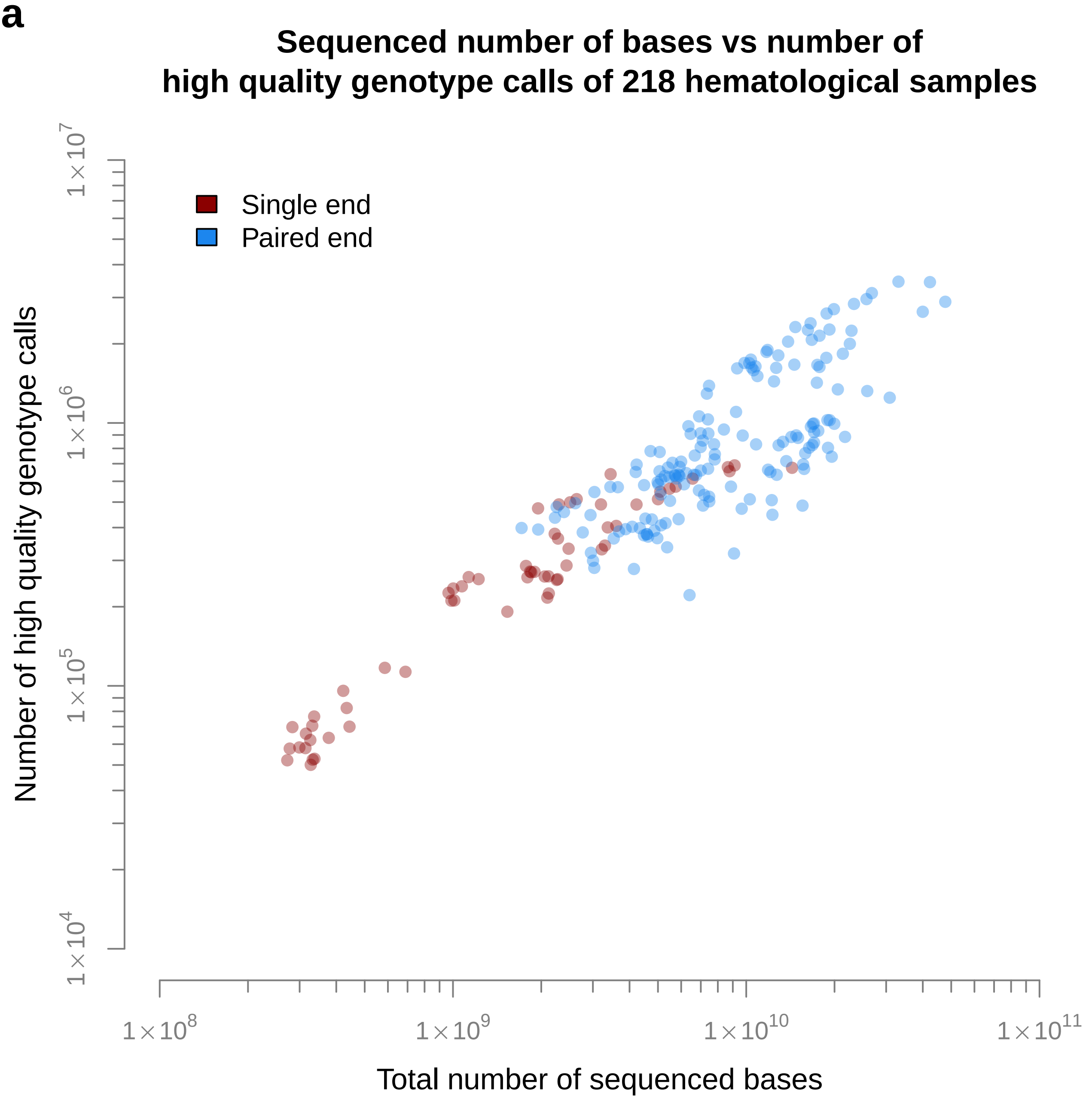
Sequenced number of bases vs number of
high quality genotype calls of 218 hematological samples
Single end
Paired end
Number of high quality genotype calls
1×10^{7}
1×10^{6}
1×10^{5}
1×10^{4}
1×10^{8}
1×10^{9}
1×10^{10}
1×10^{11}
Total number of sequenced bases

b

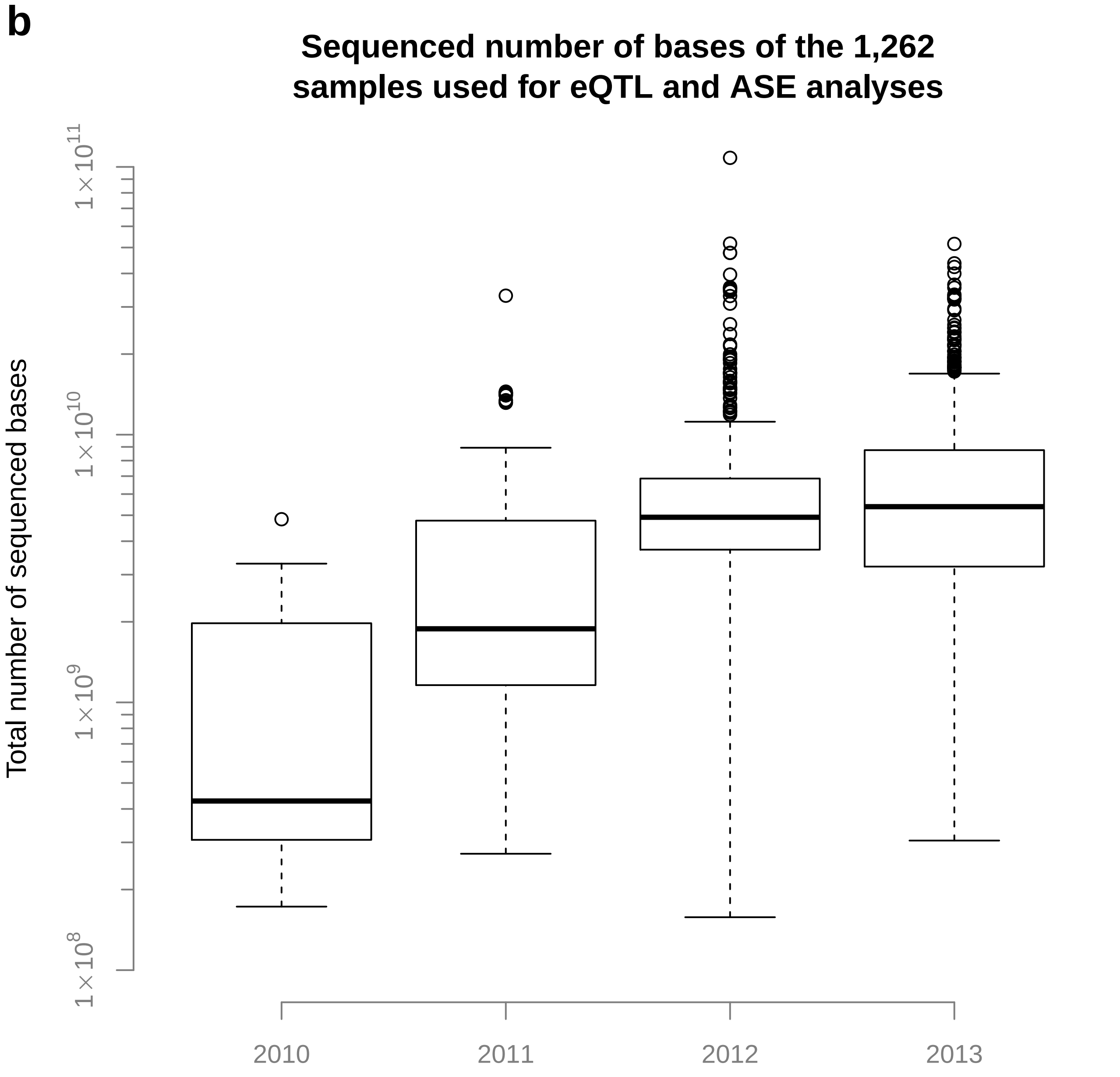
Sequenced number of bases of the 1,262
samples used for eQTL and ASE analyses
Total number of sequenced bases
1×10^{11}
1×10^{10}
1×10^{9}
1×10^{8}
2010
2011
2012
2013
