# Supplementary material for: Calling genotypes from public RNA-sequencing data enables identification of genetic variants that affect gene-expression levels
Source: Genome Med. 2015 Mar 27;7(1):30. doi: 10.1186/s13073-015-0152-4 (PMC4423486; doi:10.1186/s13073-015-0152-4)
Supplement: Additional file 8: Figure S7. — Overview of genotyping accuracy and gene expression over all chromosomes. [file 13073_2015_152_MOESM8_ESM.pdf]

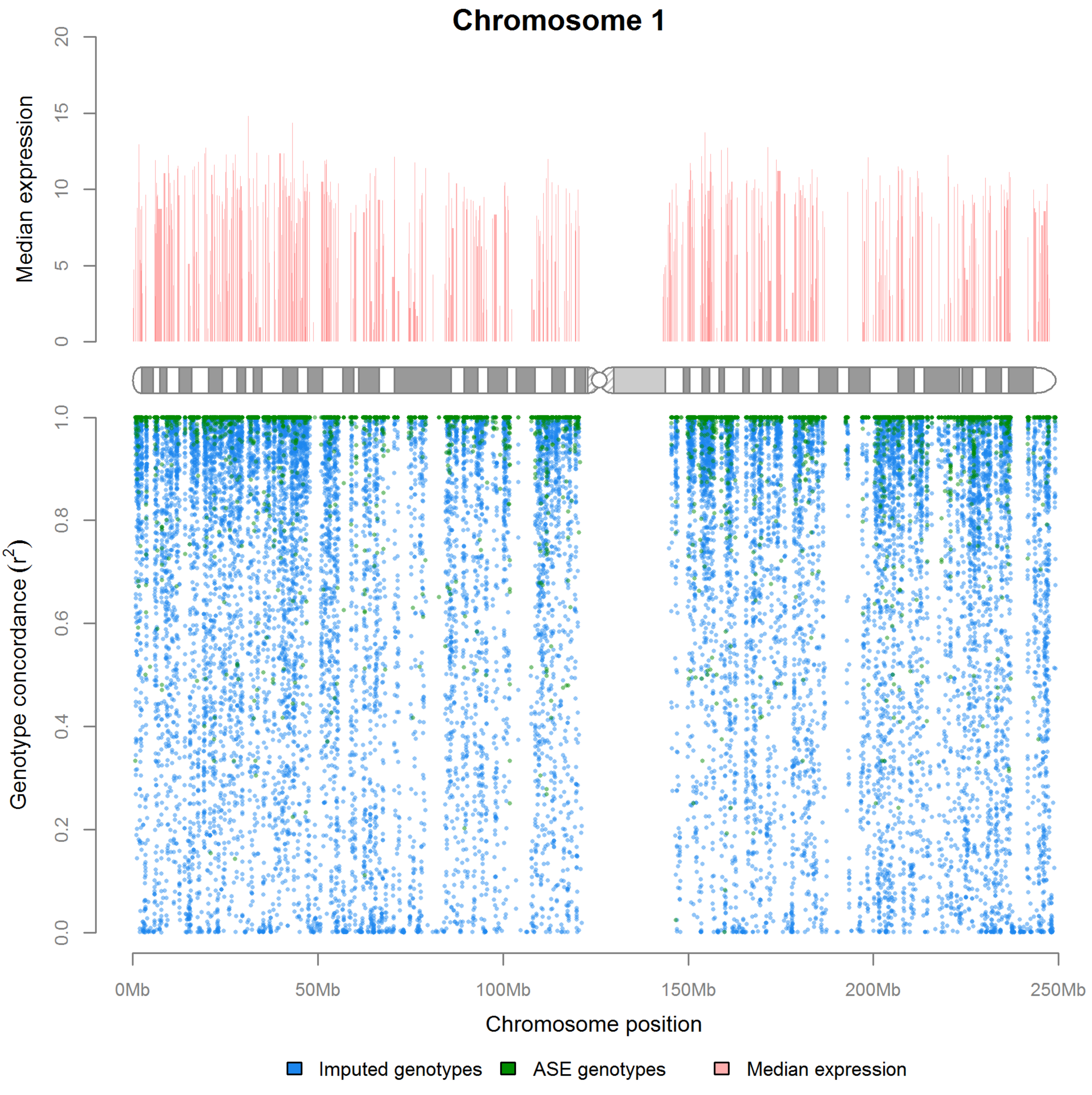

Chromosome 1
Median expression
0
5
10
15
20
Genotype concordance (r^2)
0.0
0.2
0.4
0.6
0.8
1.0
0Mb
50Mb
100Mb
150Mb
200Mb
250Mb
Chromosome position
Imputed genotypes
ASE genotypes
Median expression

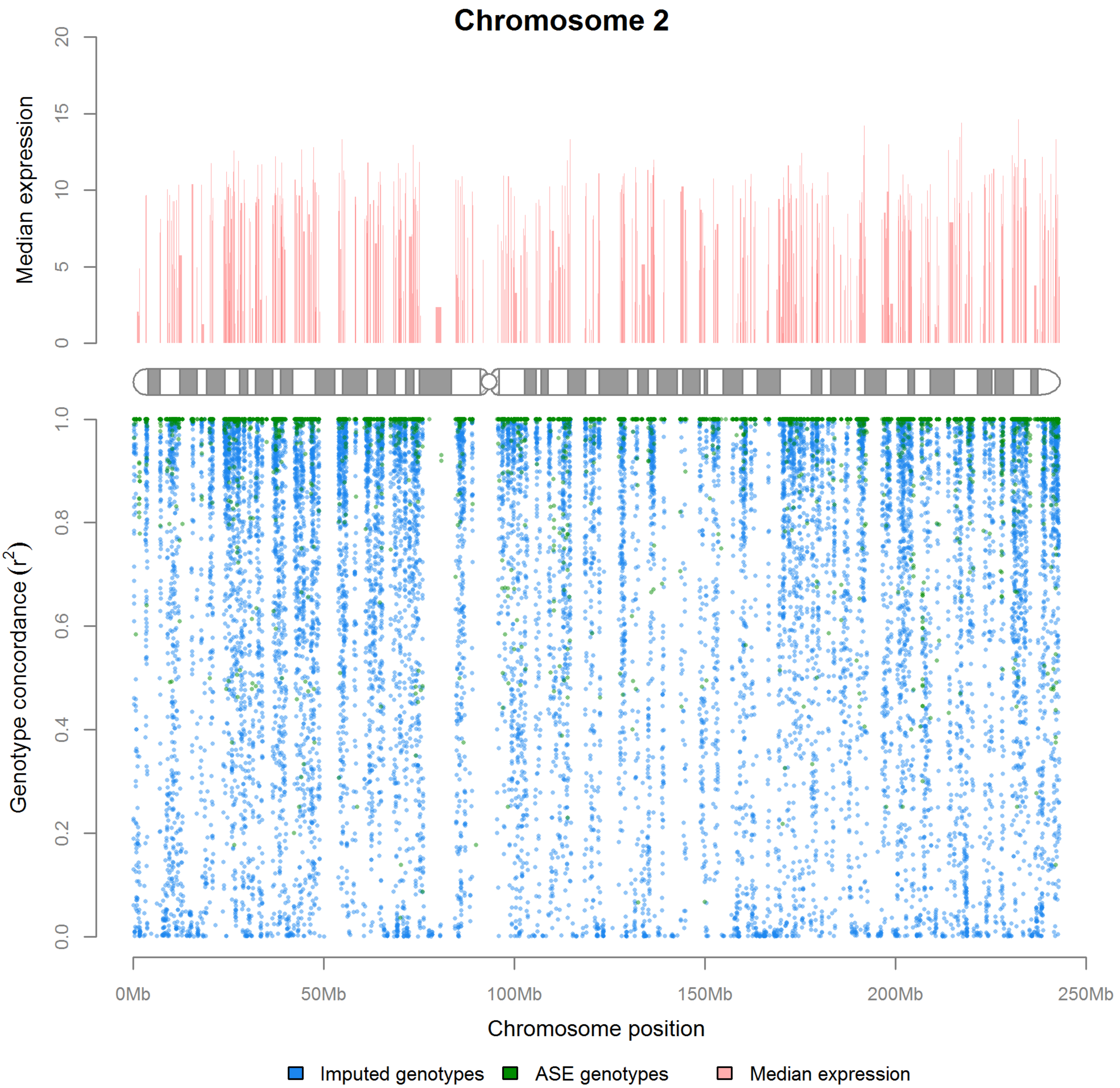

Chromosome 2
Median expression
0
5
10
15
20
Genotype concordance (r2)
0.0
0.2
0.4
0.6
0.8
1.0
0Mb
50Mb
100Mb
150Mb
200Mb
250Mb
Chromosome position
Imputed genotypes
ASE genotypes
Median expression

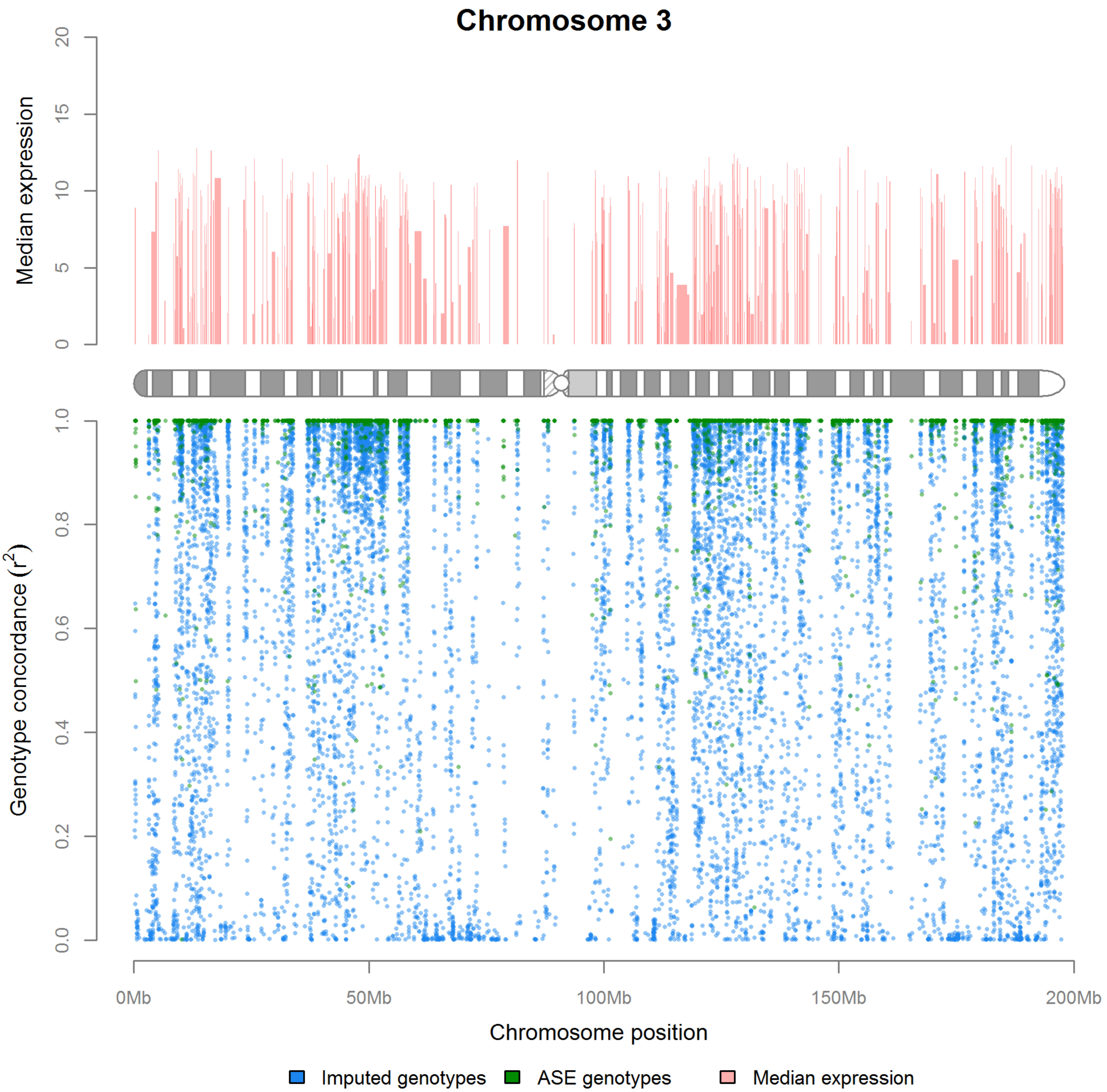

Chromosome 3
Median expression
20
15
10
5
0
Genotype concordance (r^2)
1.0
0.8
0.6
0.4
0.2
0.0
0Mb
50Mb
100Mb
150Mb
200Mb
Chromosome position
Imputed genotypes
ASE genotypes
Median expression

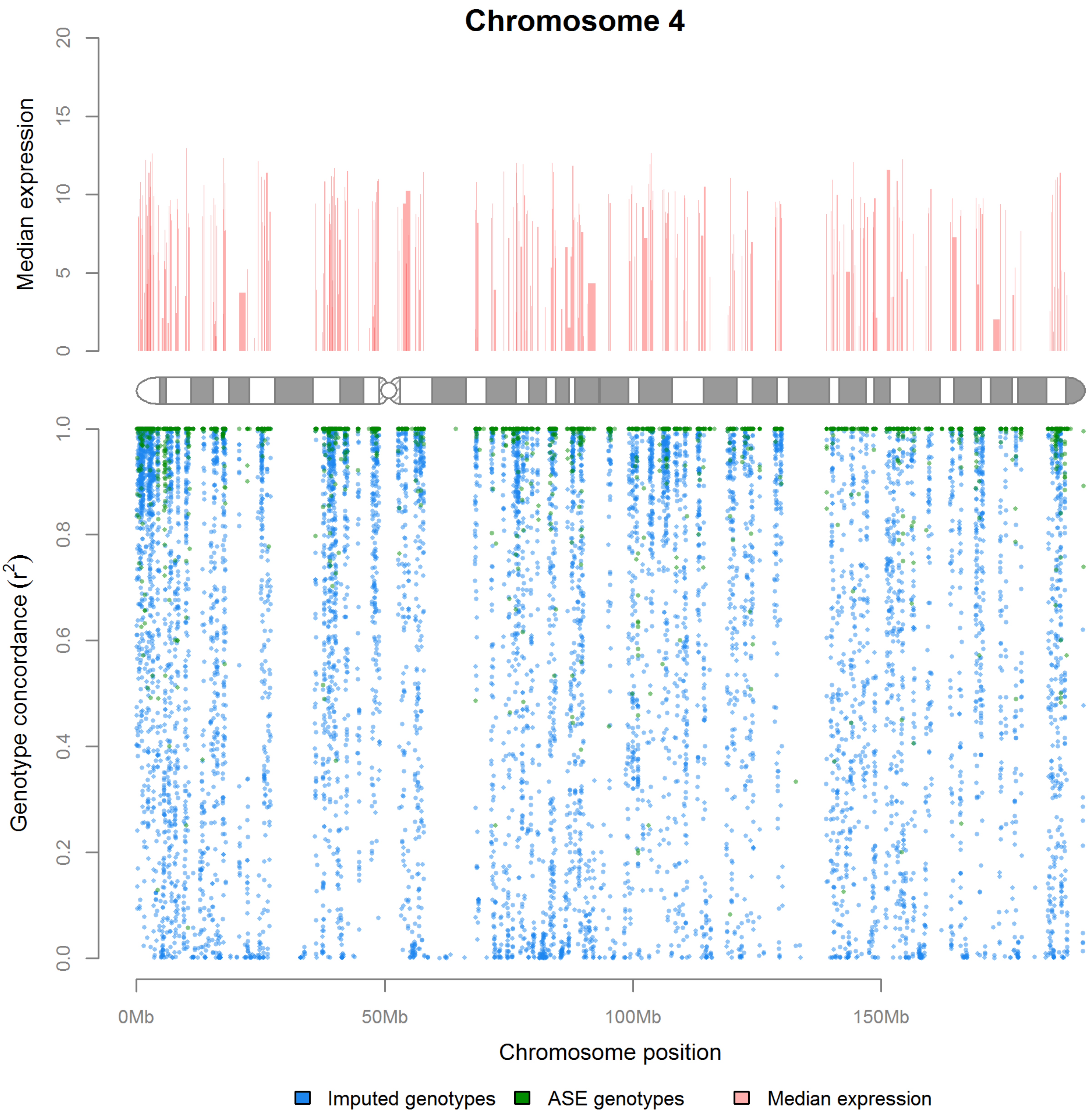

Chromosome 4
Median expression
0
5
10
15
20
Genotype concordance (r^2)
0.0
0.2
0.4
0.6
0.8
1.0
0Mb
50Mb
100Mb
150Mb
Chromosome position
Imputed genotypes
ASE genotypes
Median expression

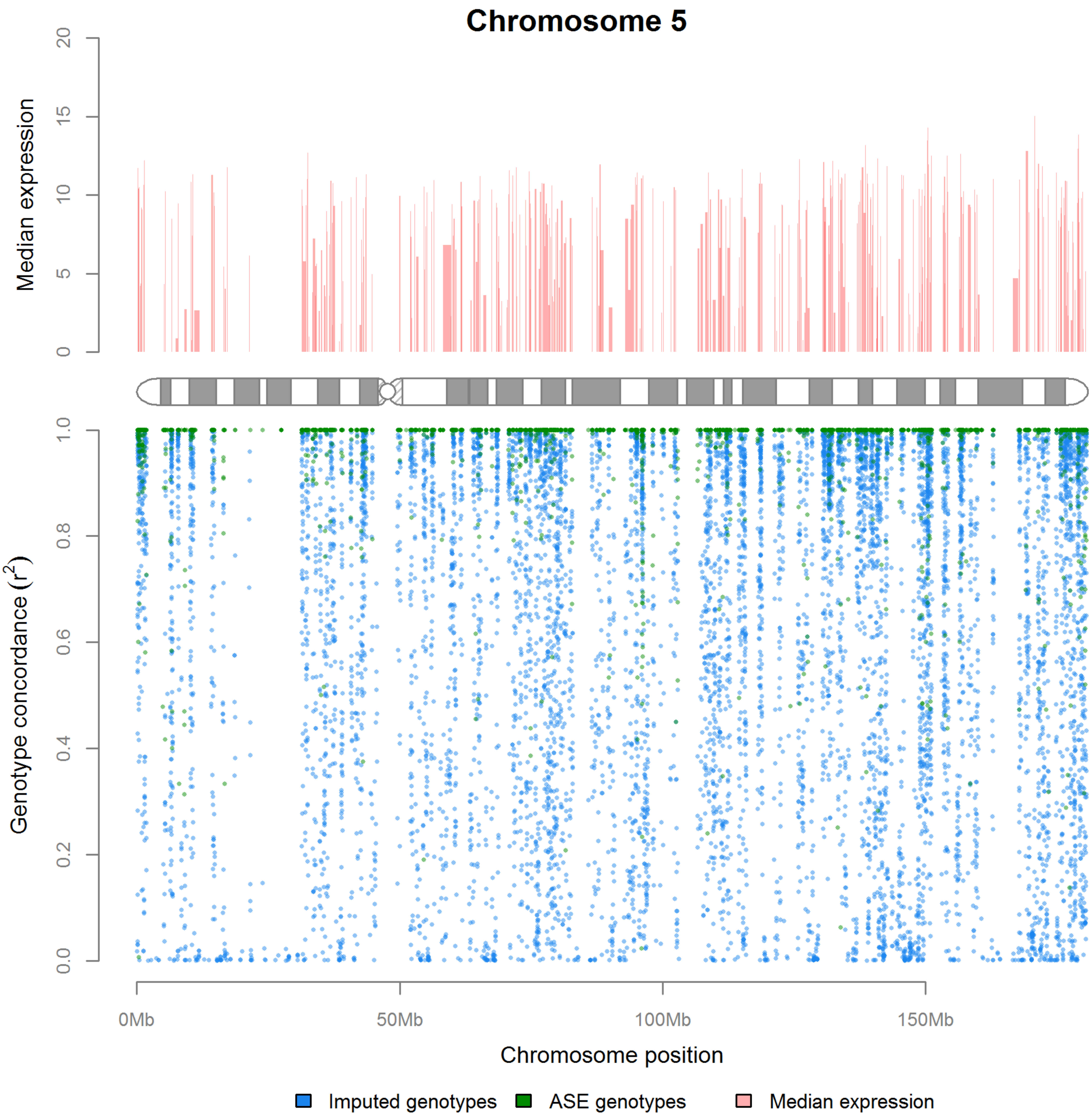

Chromosome 5
Median expression
Genotype concordance (r^2)
0
5
10
15
20
0.0
0.2
0.4
0.6
0.8
1.0
0Mb
50Mb
100Mb
150Mb
Chromosome position
Imputed genotypes
ASE genotypes
Median expression

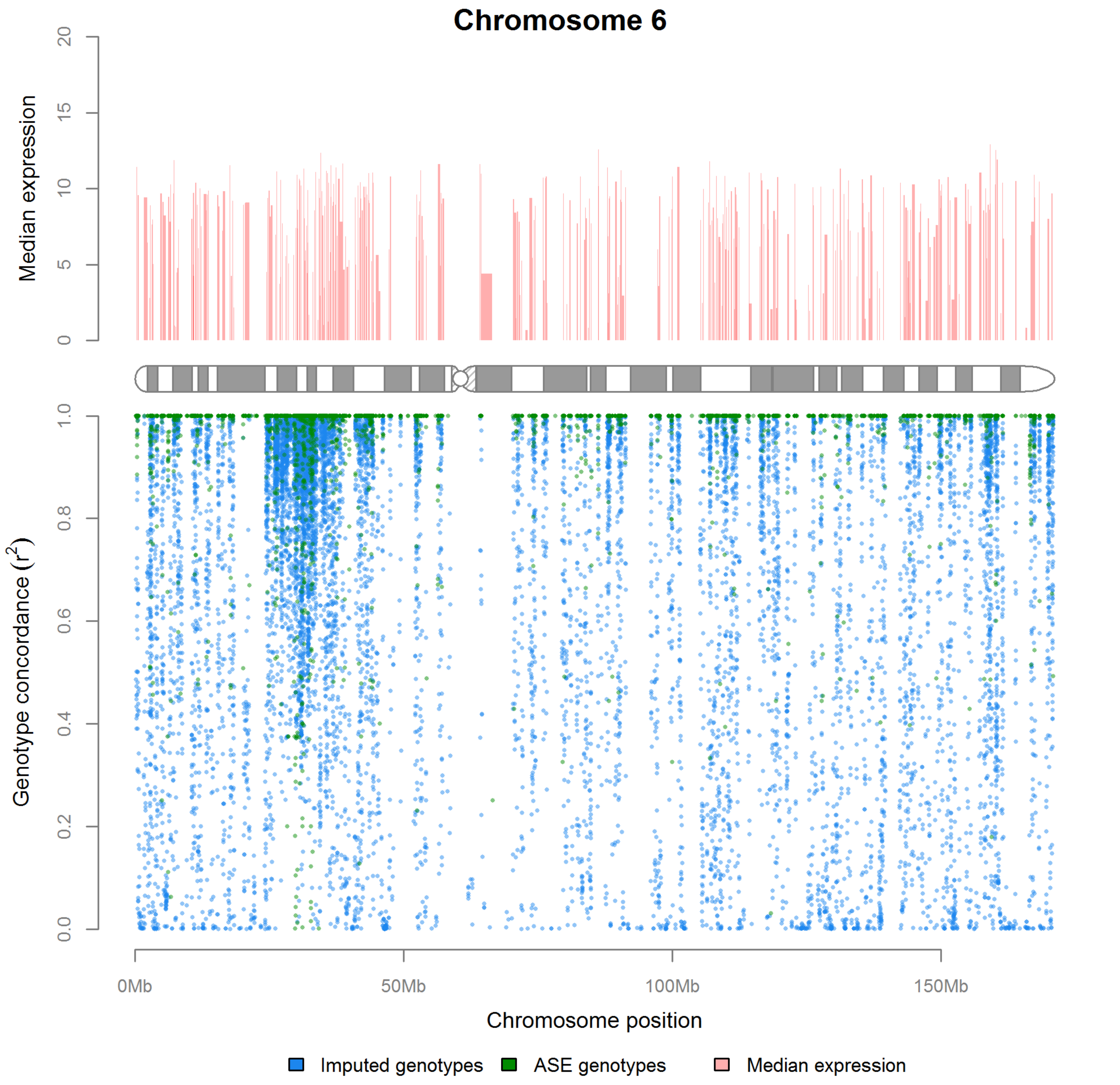

Chromosome 6
Median expression
0
5
10
15
20
Genotype concordance (r^2)
0.0
0.2
0.4
0.6
0.8
1.0
0Mb
50Mb
100Mb
150Mb
Chromosome position
Imputed genotypes
ASE genotypes
Median expression

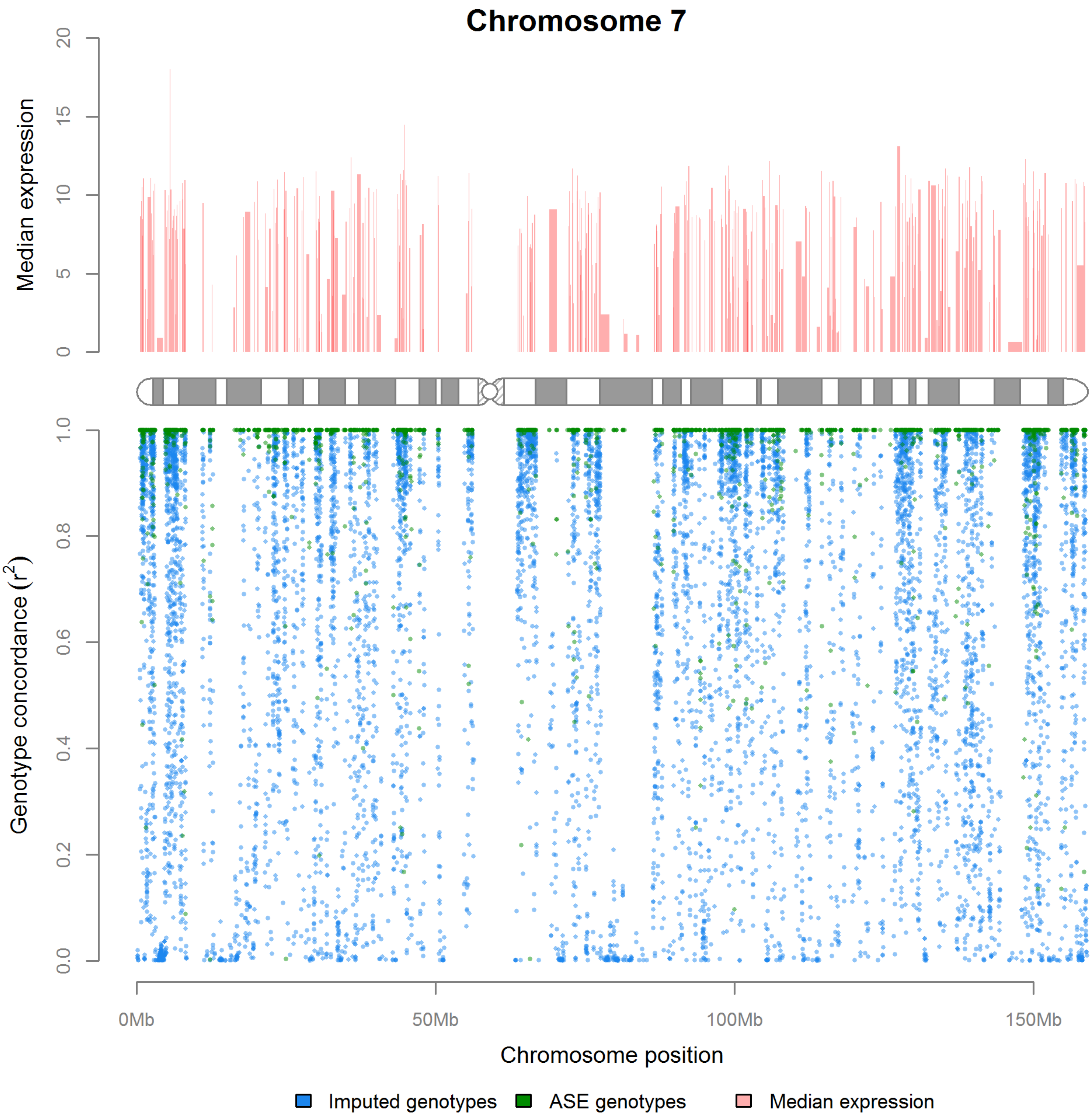

Chromosome 7
Median expression
0
5
10
15
20
Genotype concordance (r^2)
0.0
0.2
0.4
0.6
0.8
1.0
0Mb
50Mb
100Mb
150Mb
Chromosome position
Imputed genotypes
ASE genotypes
Median expression

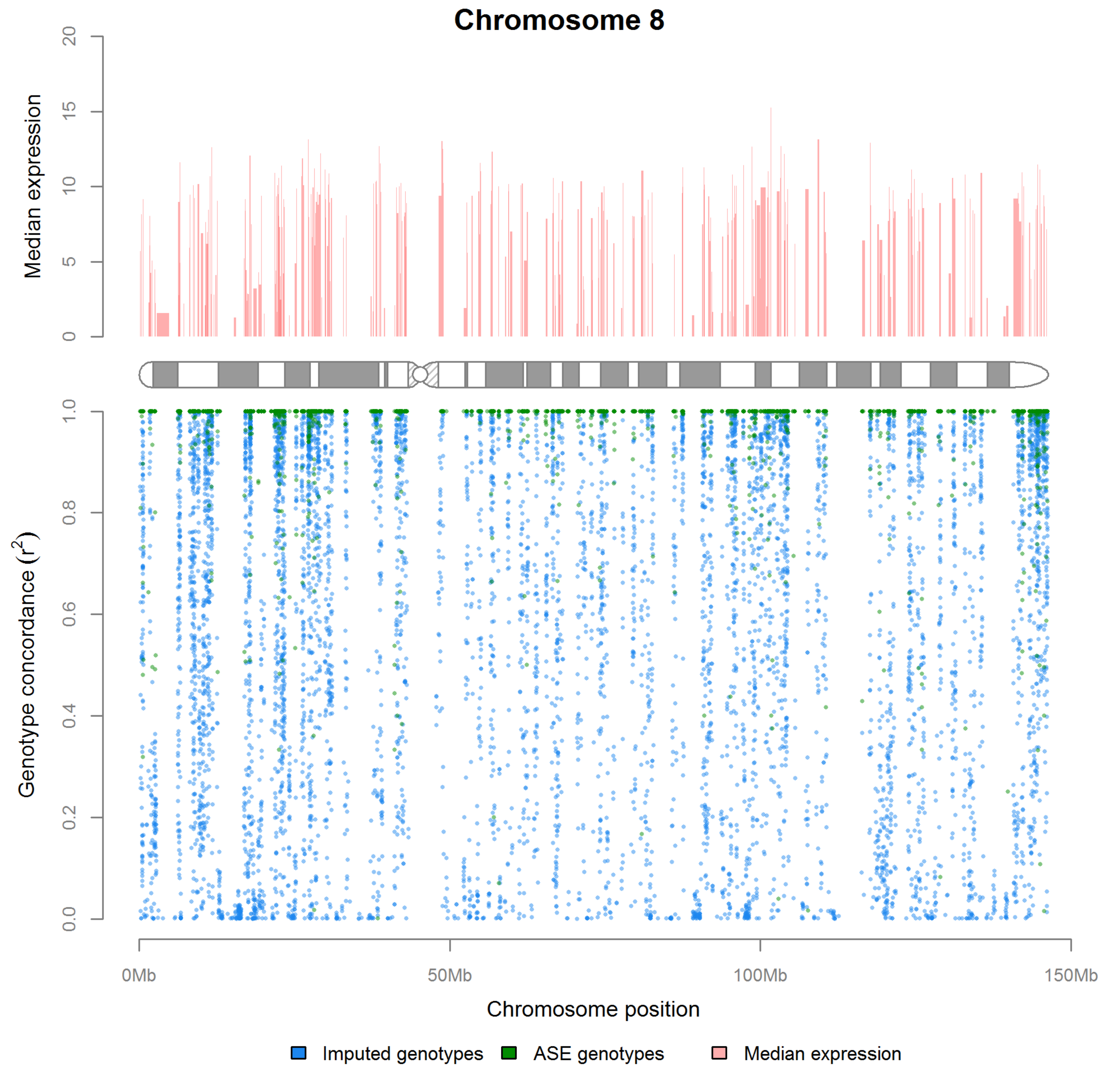

Chromosome 8
Median expression
0
5
10
15
20
Genotype concordance (r^2)
0.0
0.2
0.4
0.6
0.8
1.0
0Mb
50Mb
100Mb
150Mb
Chromosome position
Imputed genotypes
ASE genotypes
Median expression

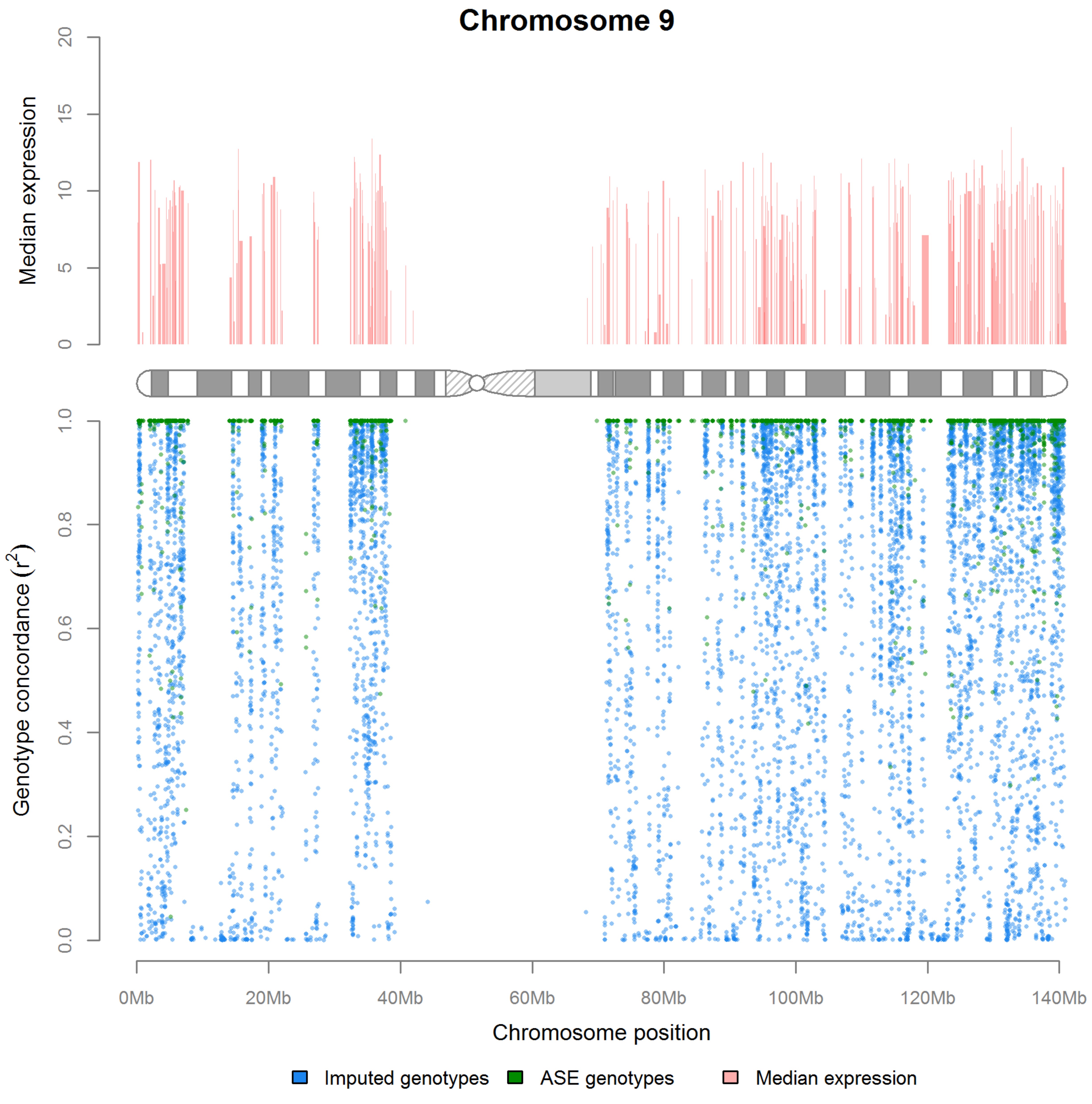

Chromosome 9
Median expression
20
15
10
5
0
Genotype concordance (r^2)
1.0
0.8
0.6
0.4
0.2
0.0
0Mb
20Mb
40Mb
60Mb
80Mb
100Mb
120Mb
140Mb
Chromosome position
Imputed genotypes
ASE genotypes
Median expression

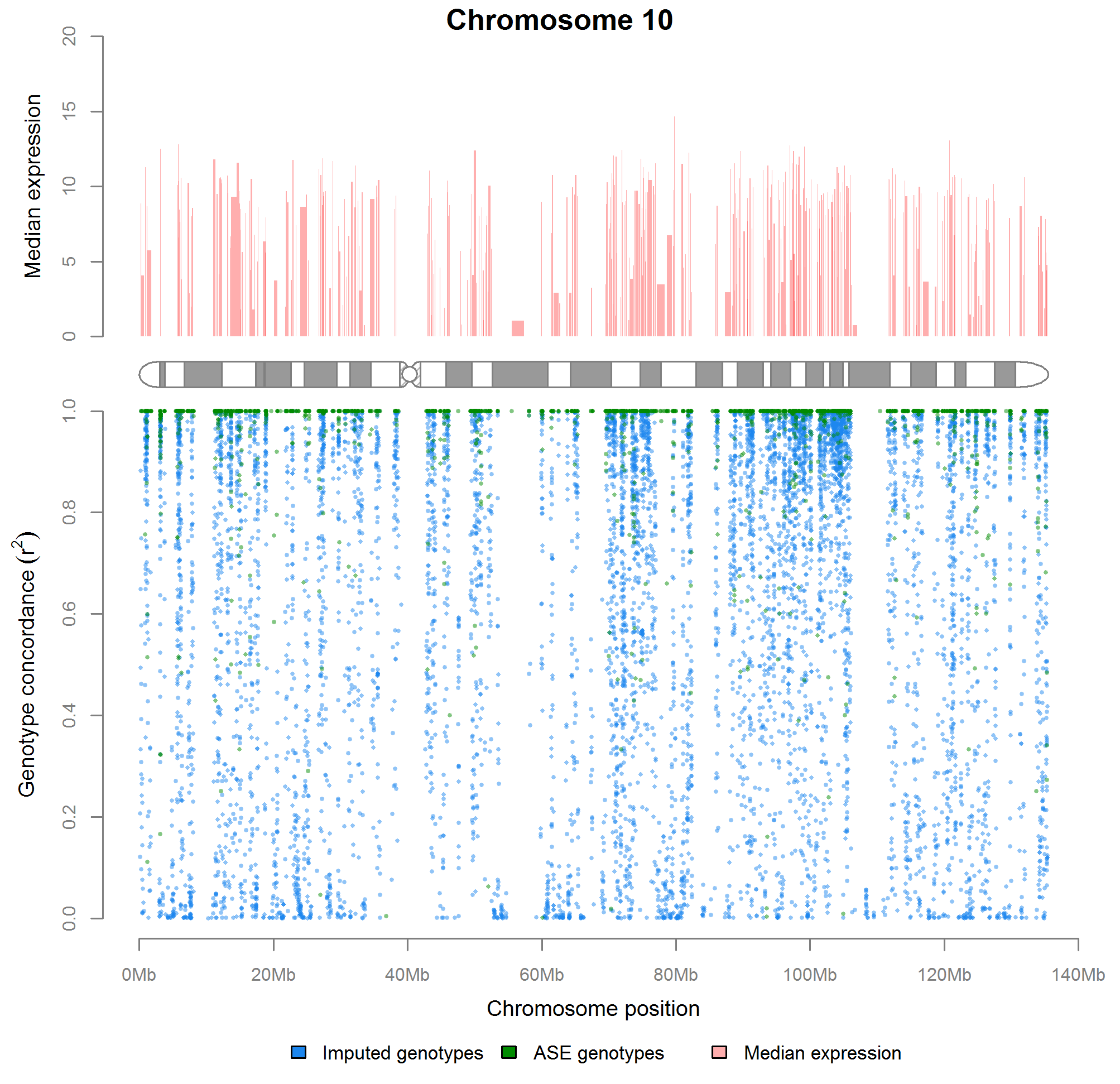
Chromosome 10
Median expression
0
5
10
15
20
Genotype concordance (r^2)
0.0
0.2
0.4
0.6
0.8
1.0
0Mb
20Mb
40Mb
60Mb
80Mb
100Mb
120Mb
140Mb
Chromosome position
Imputed genotypes
ASE genotypes
Median expression

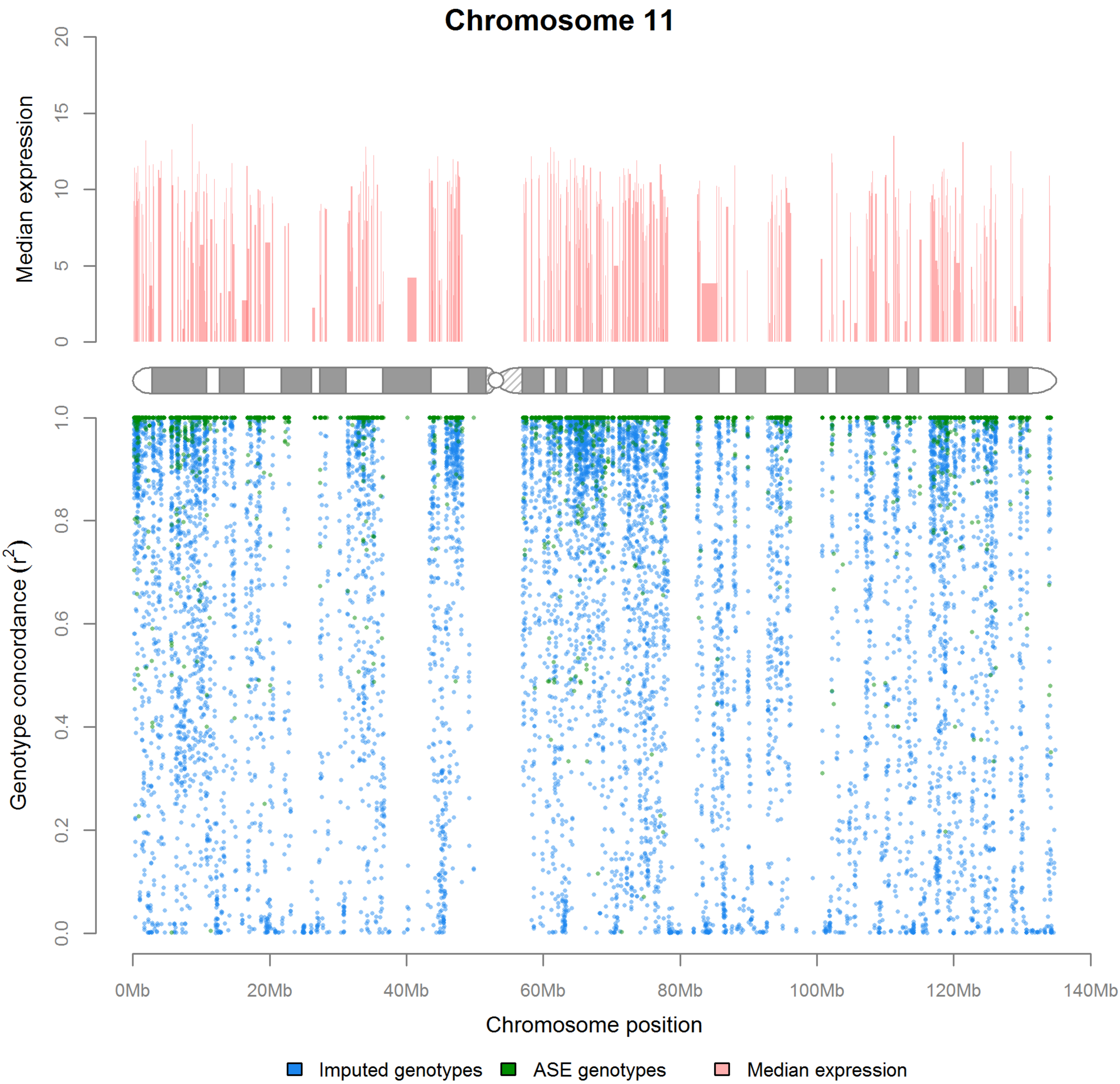

Chromosome 11
Median expression
0
5
10
15
20
Genotype concordance (r^2)
0.0
0.2
0.4
0.6
0.8
1.0
0Mb
20Mb
40Mb
60Mb
80Mb
100Mb
120Mb
140Mb
Chromosome position
Imputed genotypes
ASE genotypes
Median expression

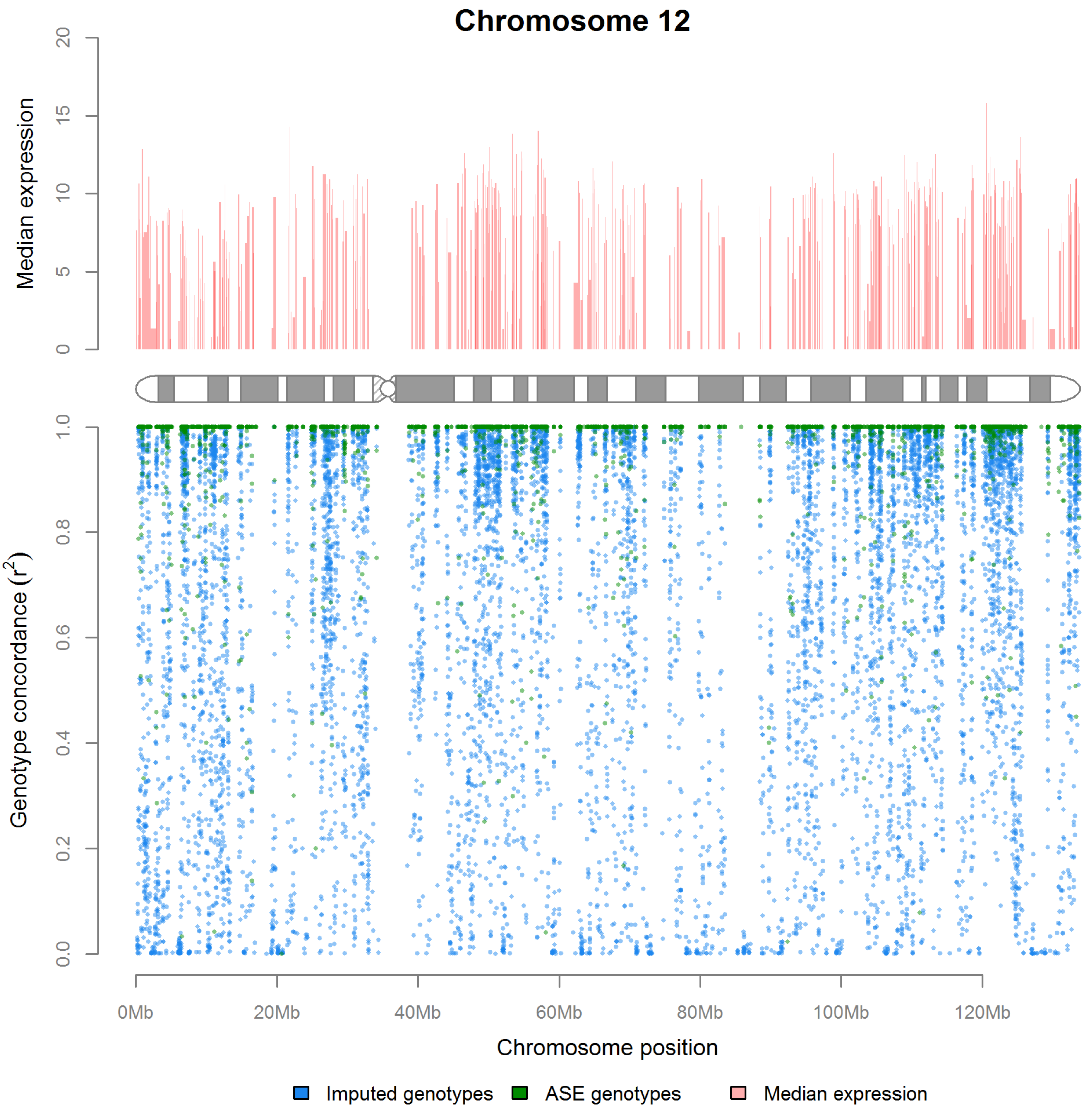

Chromosome 12
Median expression
0
5
10
15
20
1.0
0.8
0.6
0.4
0.2
0.0
Genotype concordance (r^2)
0Mb
20Mb
40Mb
60Mb
80Mb
100Mb
120Mb
Chromosome position
Imputed genotypes
ASE genotypes
Median expression

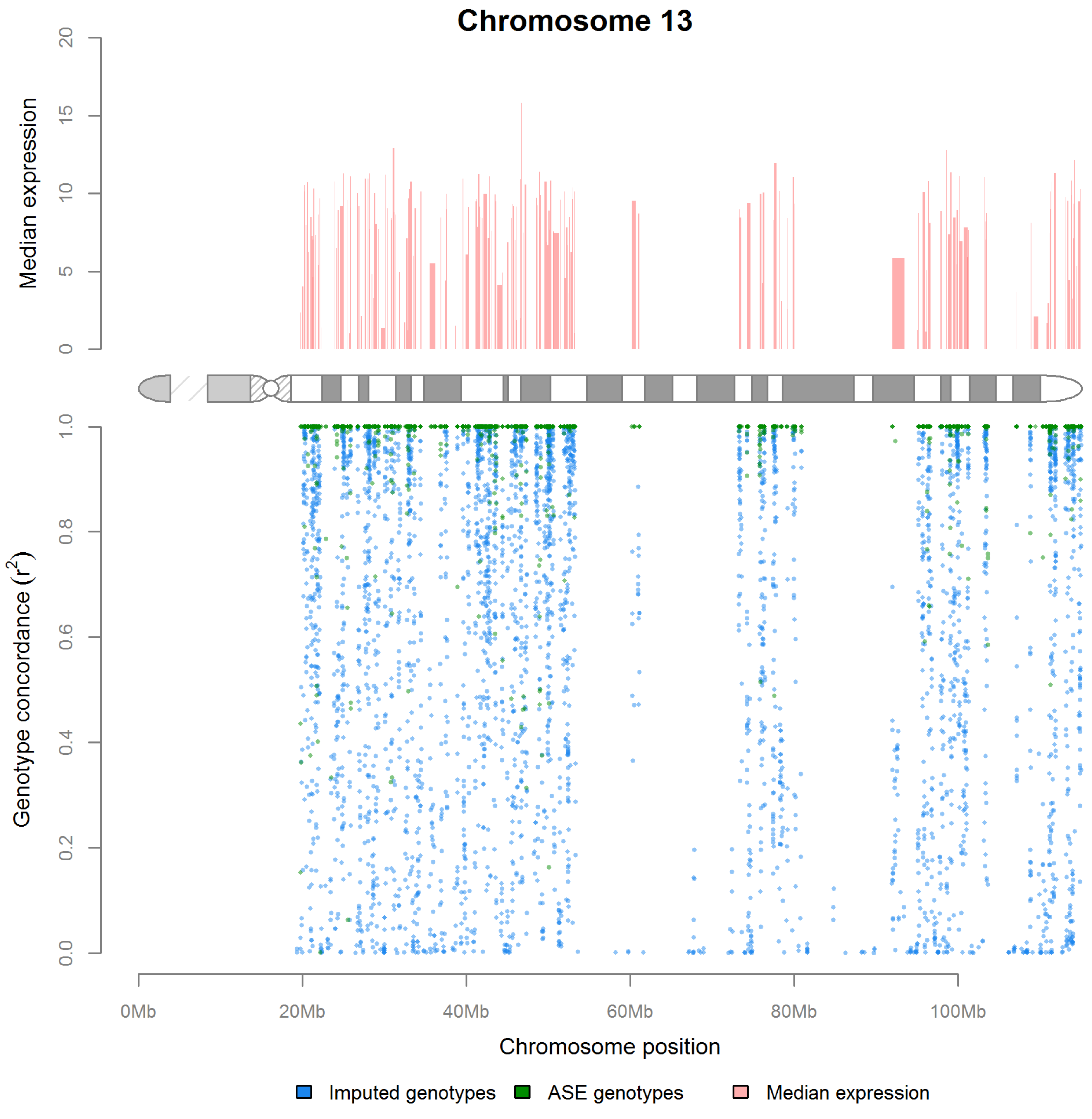
Chromosome 13
Median expression
0
5
10
15
20
Genotype concordance (r^2)
0.0
0.2
0.4
0.6
0.8
1.0
0Mb
20Mb
40Mb
60Mb
80Mb
100Mb
Chromosome position
Imputed genotypes
ASE genotypes
Median expression

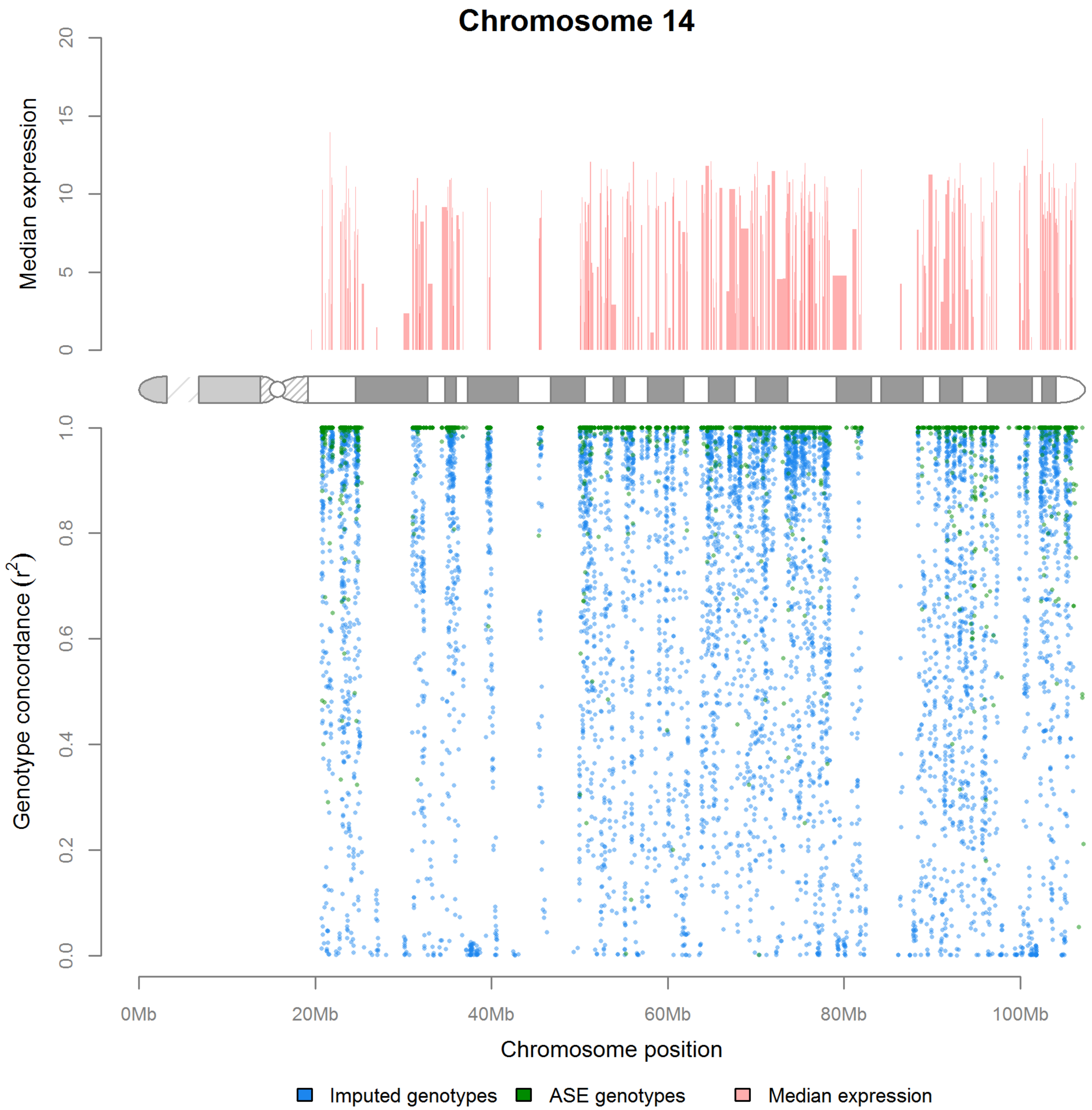

Chromosome 14
Median expression
0
5
10
15
20
Genotype concordance (r^2)
0.0
0.2
0.4
0.6
0.8
1.0
0Mb
20Mb
40Mb
60Mb
80Mb
100Mb
Chromosome position
Imputed genotypes
ASE genotypes
Median expression

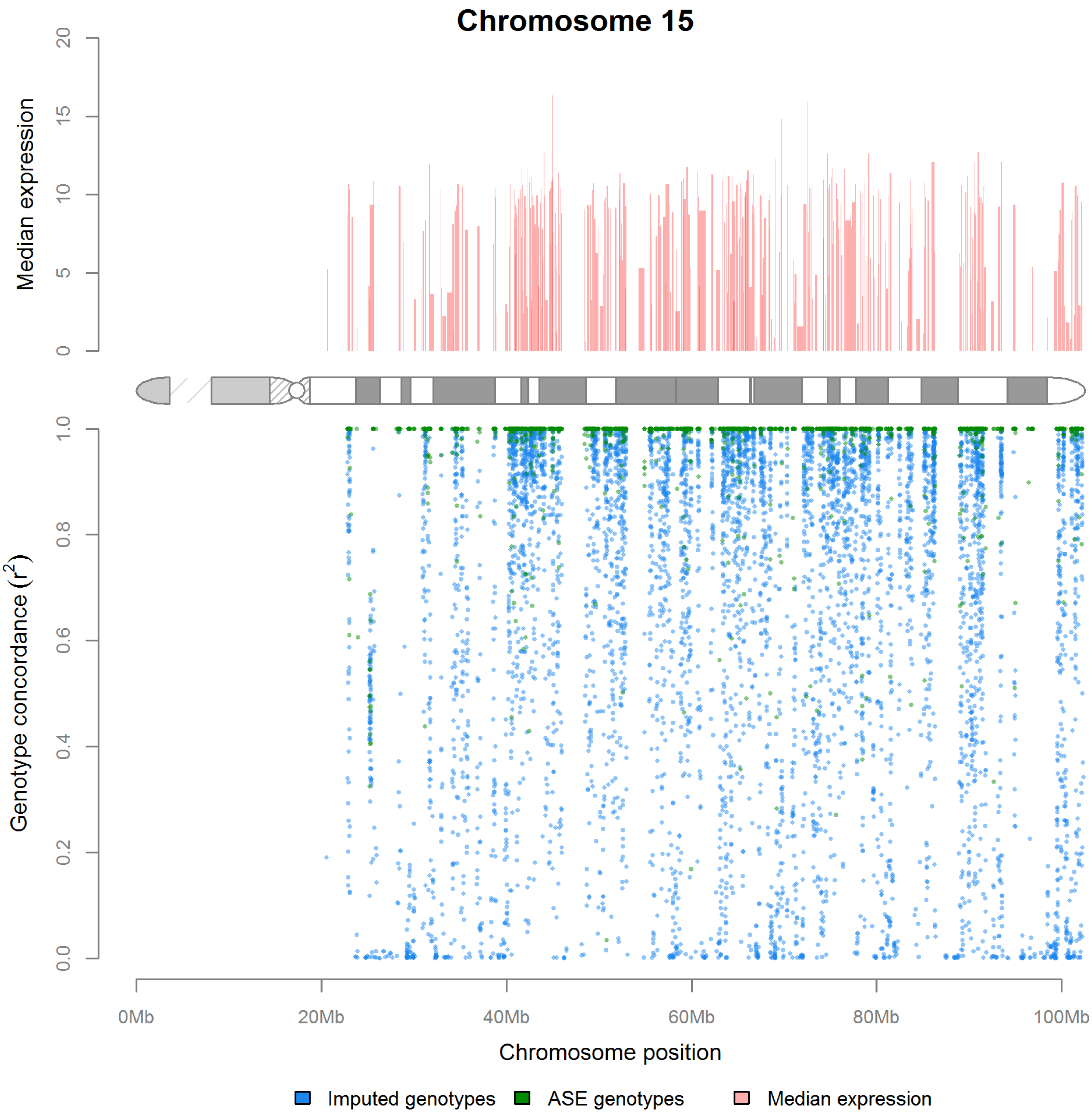

Chromosome 15
Median expression
0
5
10
15
20
Genotype concordance (r^2)
0.0
0.2
0.4
0.6
0.8
1.0
0Mb
20Mb
40Mb
60Mb
80Mb
100Mb
Chromosome position
Imputed genotypes
ASE genotypes
Median expression

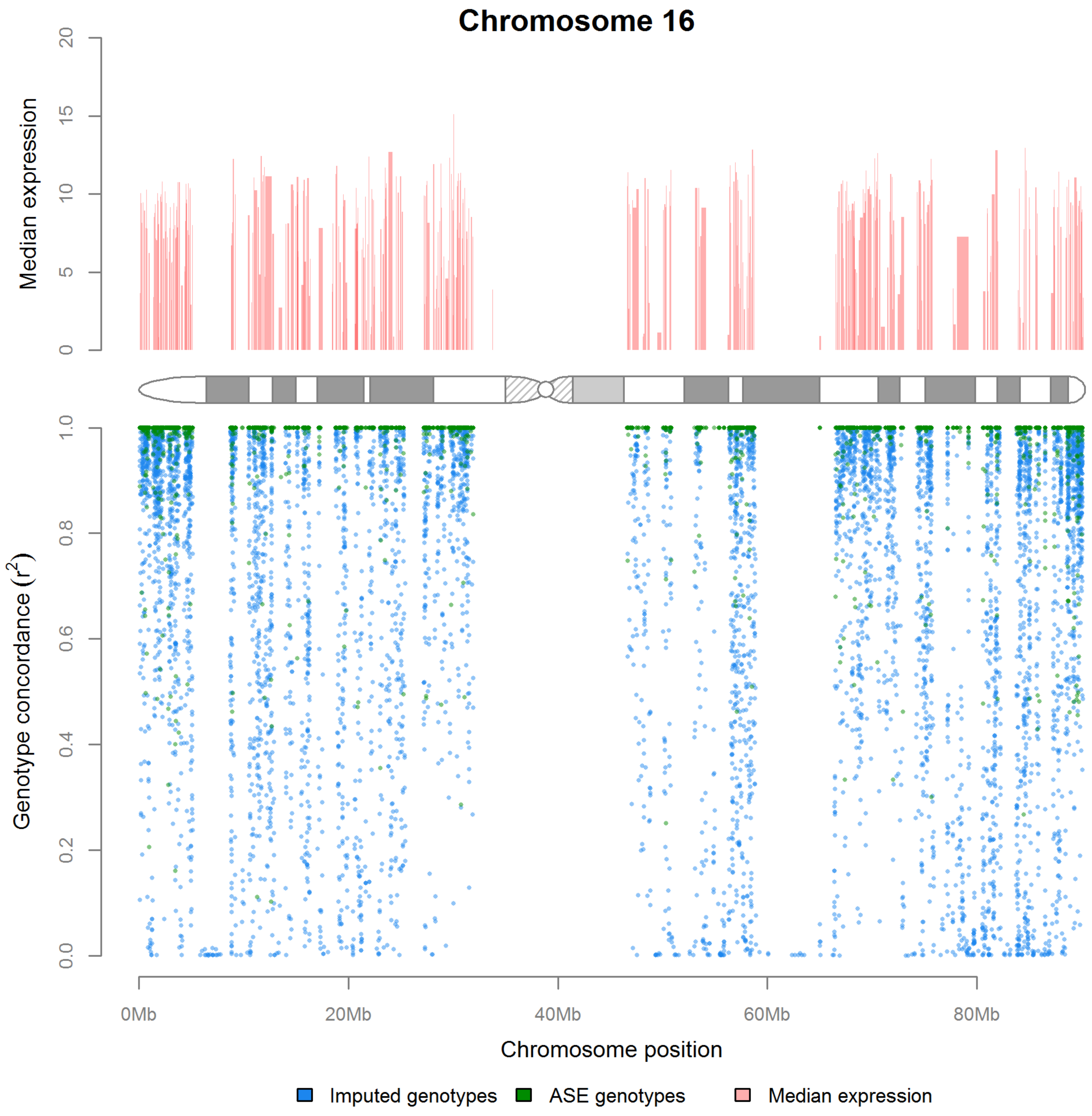
Chromosome 16
Median expression
0
5
10
15
20
Genotype concordance (r^2)
0.0
0.2
0.4
0.6
0.8
1.0
0Mb
20Mb
40Mb
60Mb
80Mb
Chromosome position
Imputed genotypes
ASE genotypes
Median expression

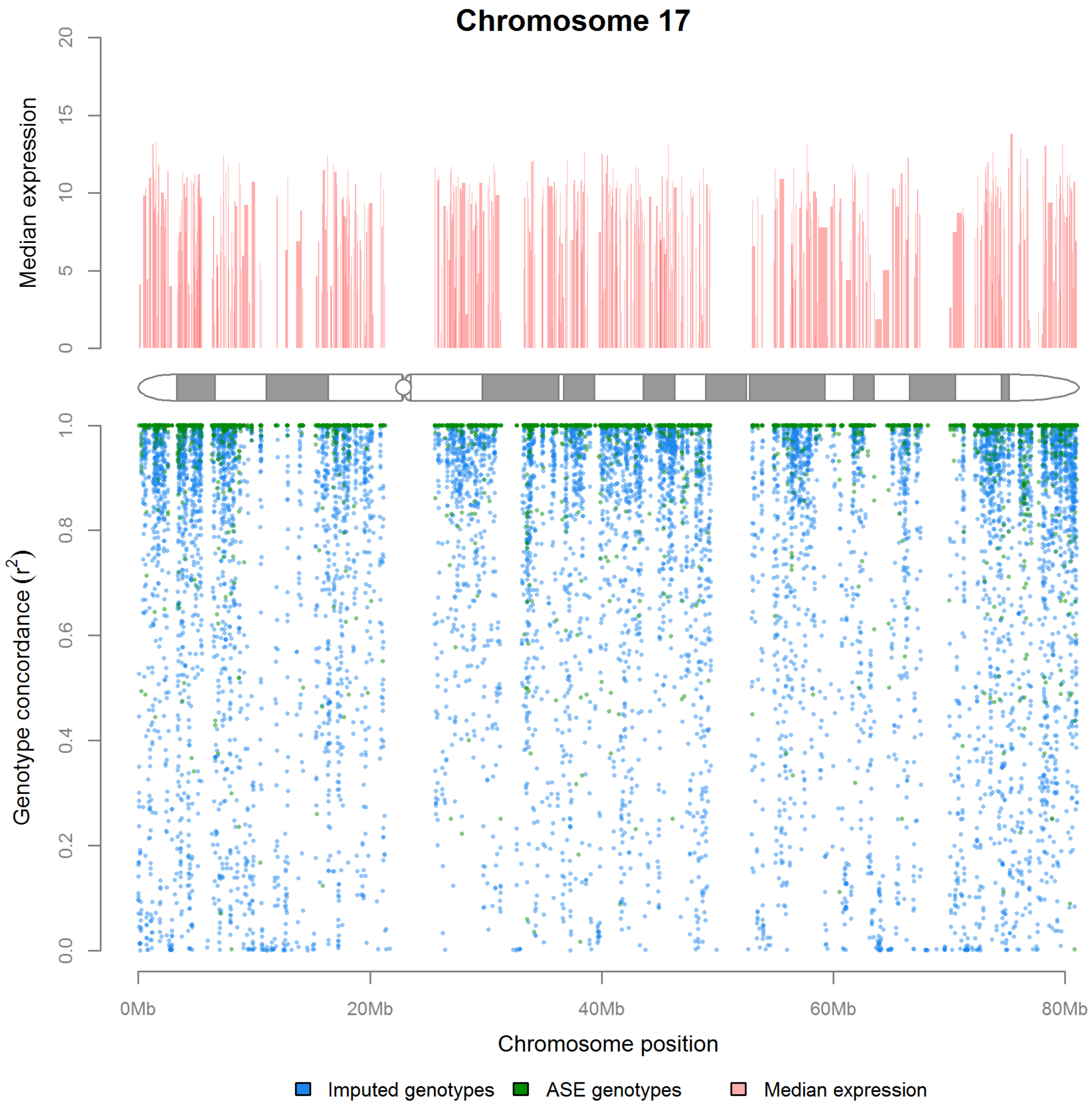

Chromosome 17
Median expression
0
5
10
15
20
Genotype concordance (r^2)
0.0
0.2
0.4
0.6
0.8
1.0
0Mb
20Mb
40Mb
60Mb
80Mb
Chromosome position
Imputed genotypes
ASE genotypes
Median expression

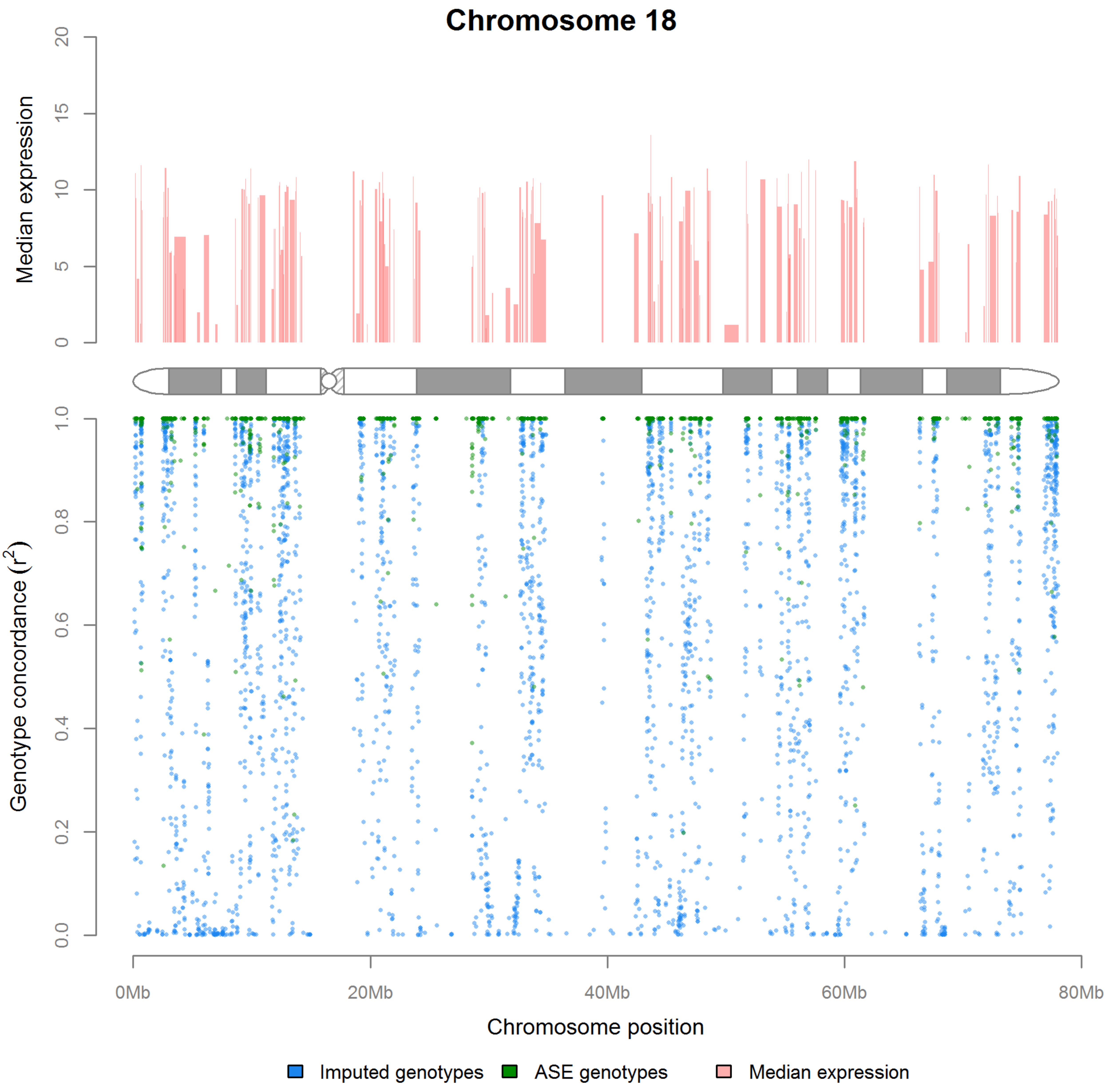

Chromosome 18
Median expression
20
15
10
5
0
Genotype concordance (r²)
1.0
0.8
0.6
0.4
0.2
0.0
0Mb
20Mb
40Mb
60Mb
80Mb
Chromosome position
Imputed genotypes
ASE genotypes
Median expression

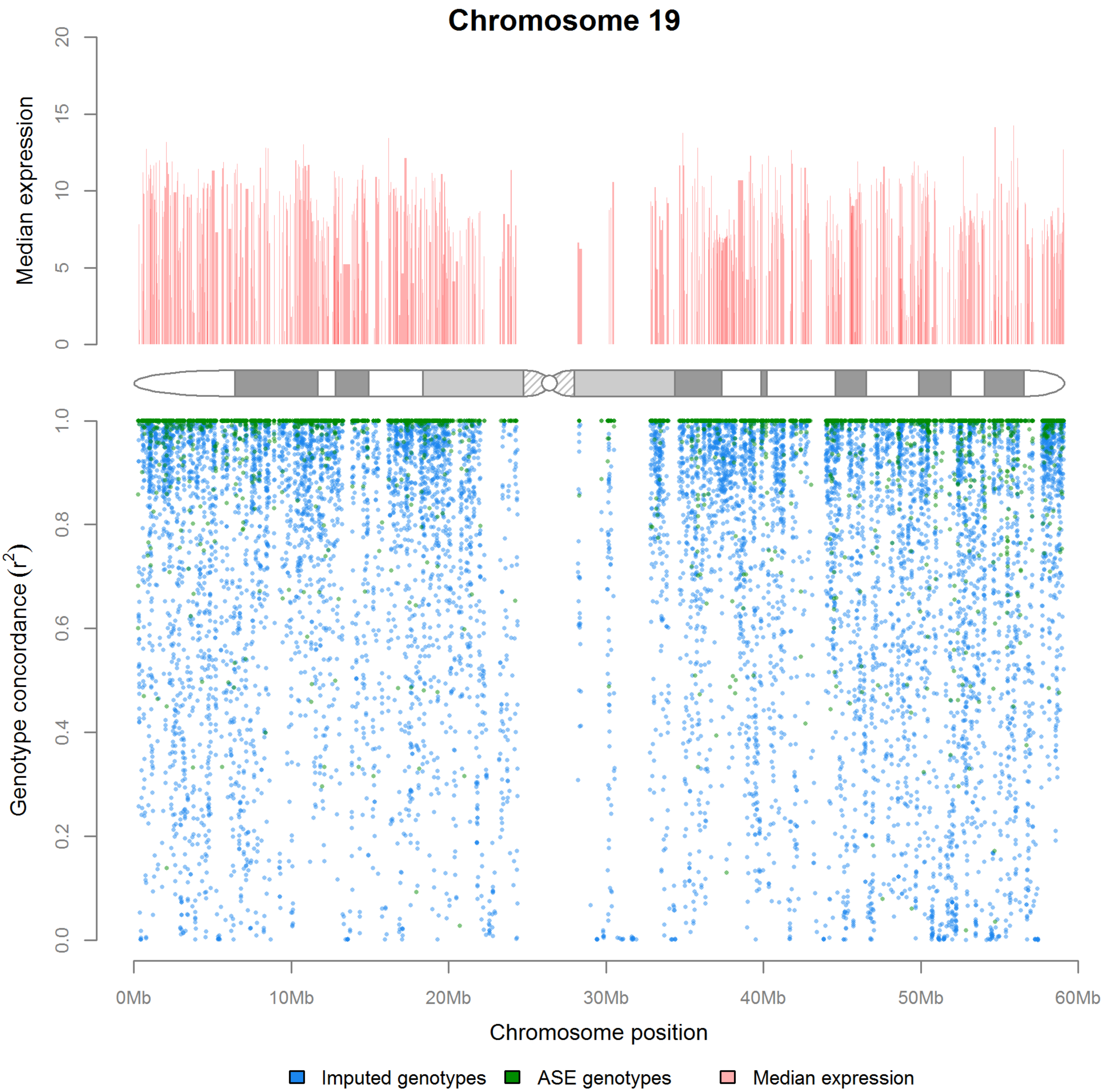

Chromosome 19
Median expression
0
5
10
15
20
Genotype concordance (r^2)
0.0
0.2
0.4
0.6
0.8
1.0
0Mb
10Mb
20Mb
30Mb
40Mb
50Mb
60Mb
Chromosome position
Imputed genotypes
ASE genotypes
Median expression

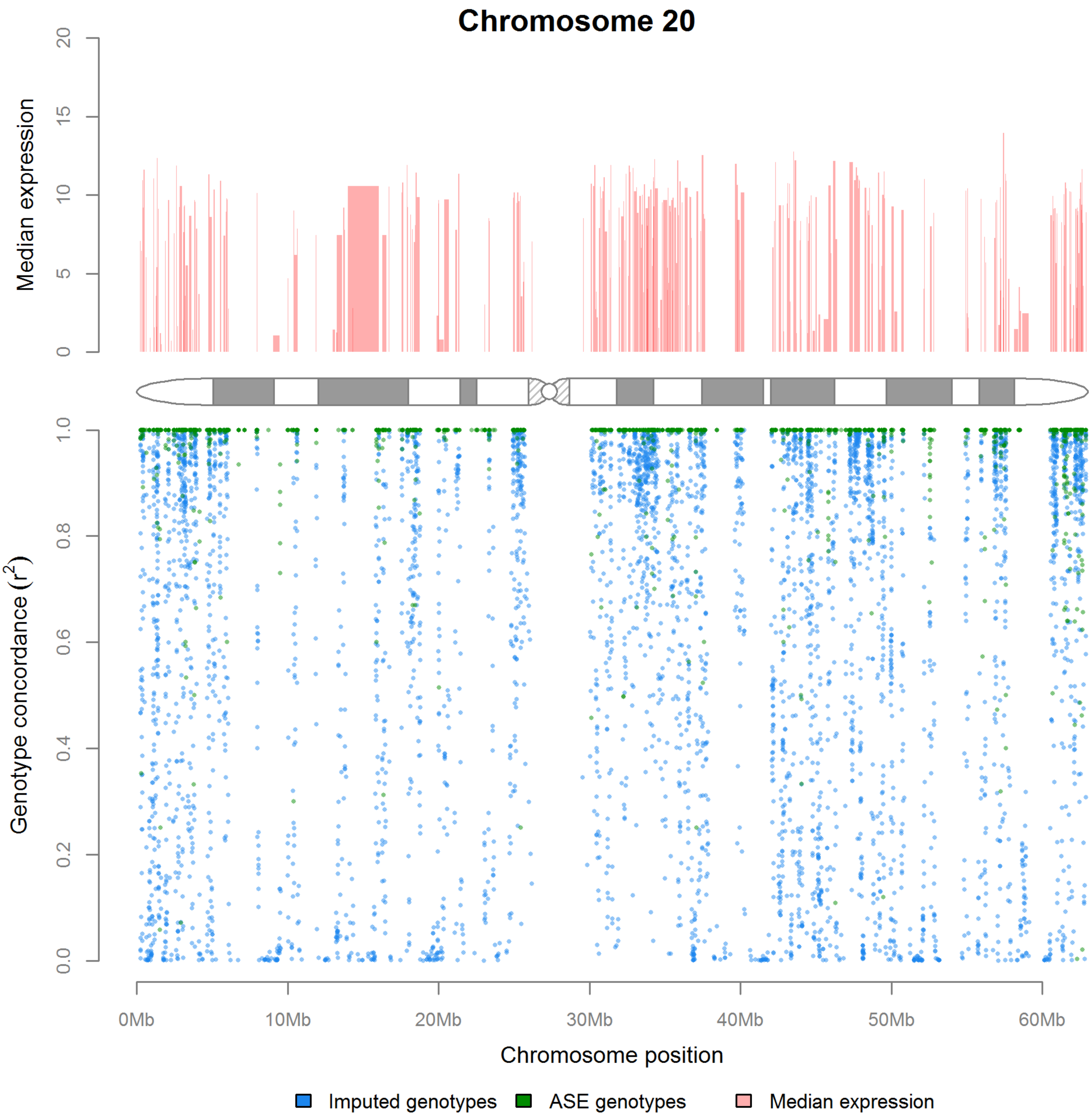

Chromosome 20
Median expression
0
5
10
15
20
Genotype concordance (r^2)
0.0
0.2
0.4
0.6
0.8
1.0
0Mb
10Mb
20Mb
30Mb
40Mb
50Mb
60Mb
Chromosome position
Imputed genotypes
ASE genotypes
Median expression

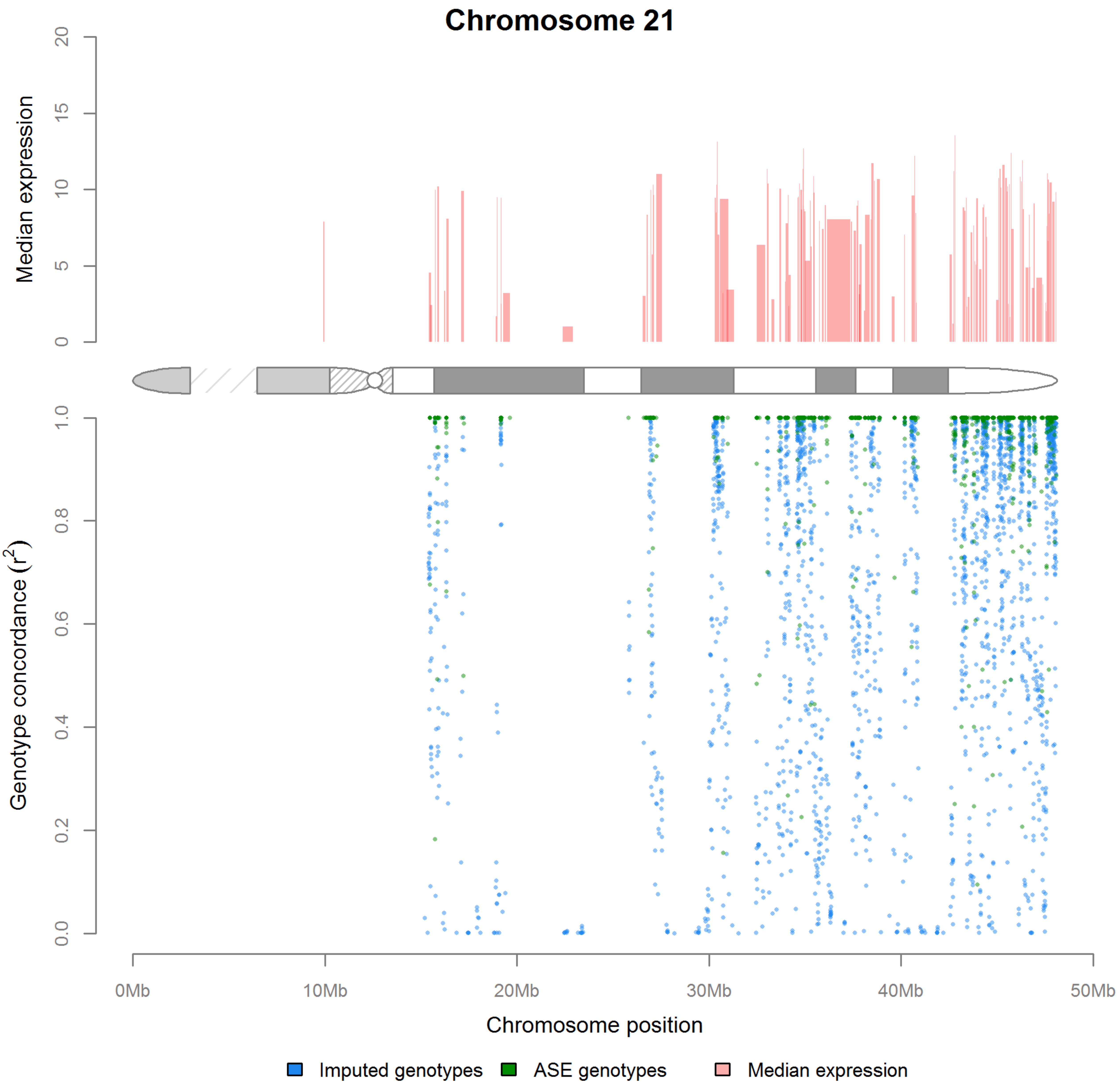

Chromosome 21
Median expression
0
5
10
15
20
Genotype concordance (r^2)
0.0
0.2
0.4
0.6
0.8
1.0
0Mb
10Mb
20Mb
30Mb
40Mb
50Mb
Chromosome position
Imputed genotypes
ASE genotypes
Median expression

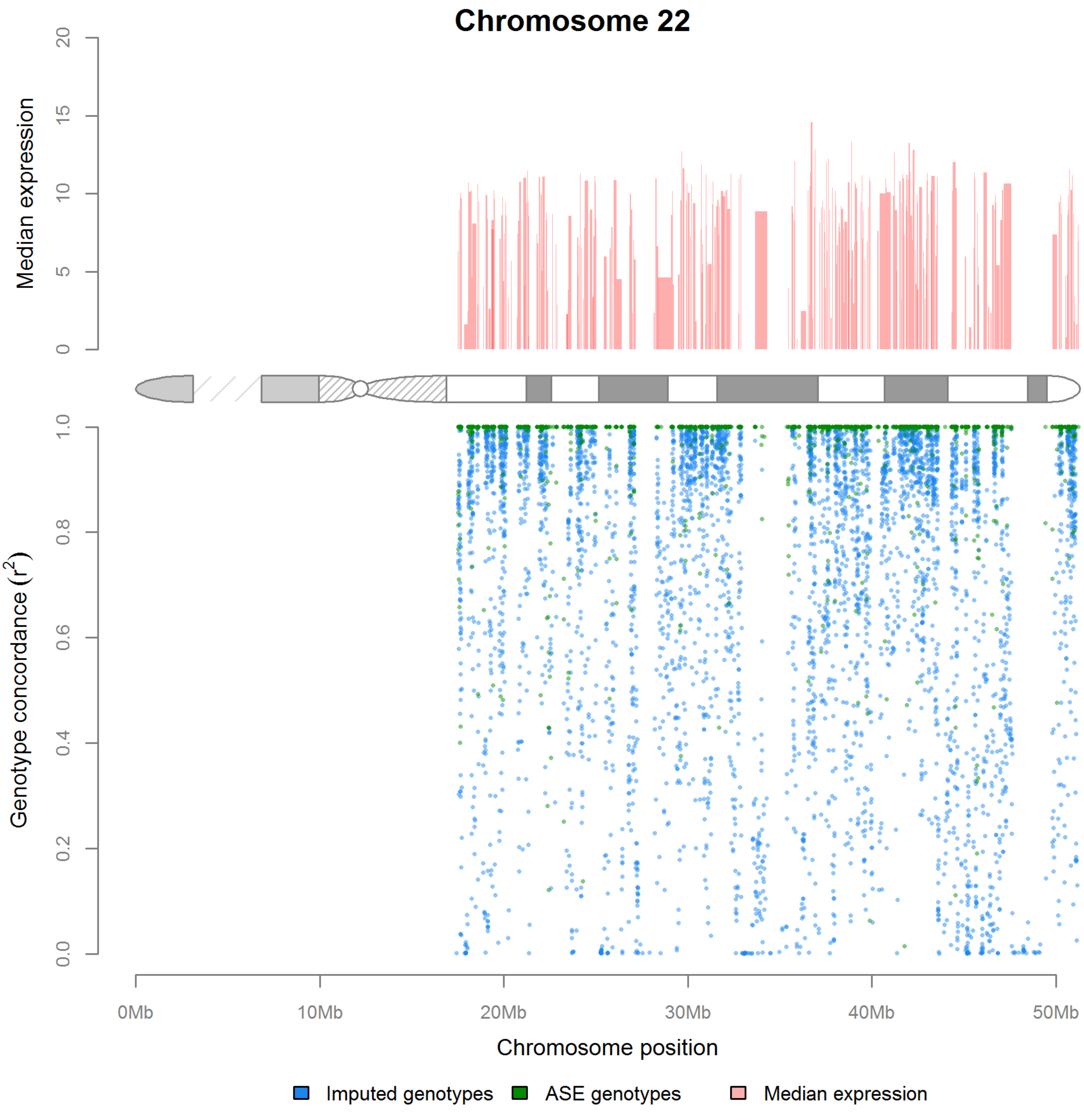
Chromosome 22
Median expression
0
5
10
15
20
Genotype concordance (r^2)
0.0
0.2
0.4
0.6
0.8
1.0
0Mb
10Mb
20Mb
30Mb
40Mb
50Mb
Chromosome position
Imputed genotypes
ASE genotypes
Median expression
