# Supplementary material for: Calling genotypes from public RNA-sequencing data enables identification of genetic variants that affect gene-expression levels
Source: Genome Med. 2015 Mar 27;7(1):30. doi: 10.1186/s13073-015-0152-4 (PMC4423486; doi:10.1186/s13073-015-0152-4)
Supplement: Additional file 9: Figure S8. — Relation between gene-expression levels and genotype concordance before and after imputation. Genotype concordances in all Geuvadis samples (a) and European Geuvadis samples (b) of common SNPs (MAF ≥0.05, DR2 ≥ 0.8) before and after imputation grouped by the median expression levels of their genes. [file 13073_2015_152_MOESM9_ESM.pdf]

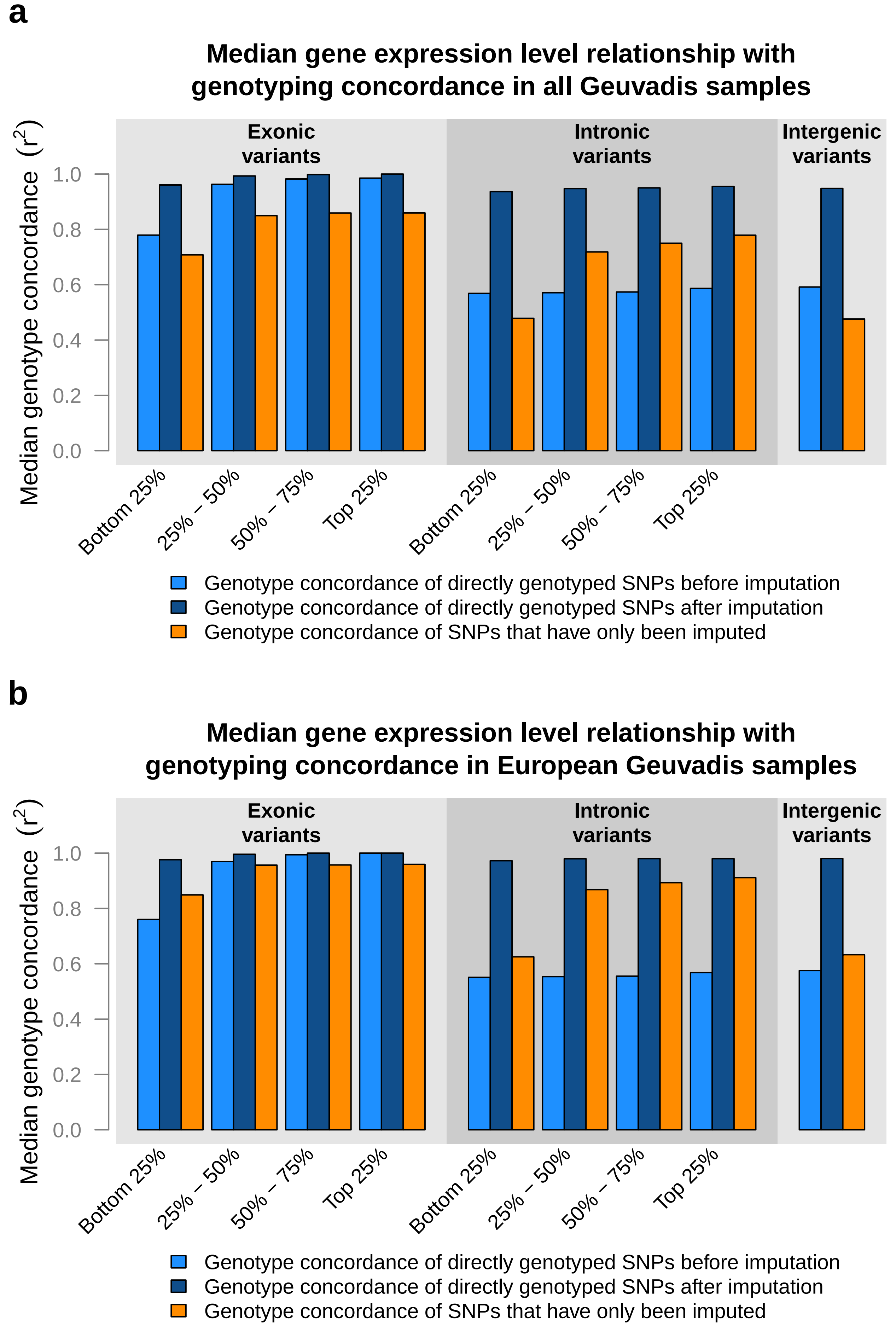
a
Median gene expression level relationship with genotyping concordance in all Geuvadis samples
Median genotype concordance (r2)
Exonic variants
Intronic variants
Intergenic variants
0.0
0.2
0.4
0.6
0.8
1.0
Bottom 25%
25% – 50%
50% – 75%
Top 25%
Genotype concordance of directly genotyped SNPs before imputation
Genotype concordance of directly genotyped SNPs after imputation
Genotype concordance of SNPs that have only been imputed
b
Median gene expression level relationship with genotyping concordance in European Geuvadis samples
Median genotype concordance (r2)
Exonic variants
Intronic variants
Intergenic variants
0.0
0.2
0.4
0.6
0.8
1.0
Bottom 25%
25% – 50%
50% – 75%
Top 25%
Genotype concordance of directly genotyped SNPs before imputation
Genotype concordance of directly genotyped SNPs after imputation
Genotype concordance of SNPs that have only been imputed
