# Supplementary material for: Calling genotypes from public RNA-sequencing data enables identification of genetic variants that affect gene-expression levels
Source: Genome Med. 2015 Mar 27;7(1):30. doi: 10.1186/s13073-015-0152-4 (PMC4423486; doi:10.1186/s13073-015-0152-4)
Supplement: Additional file 10: Figure S9. — Expression of tissue-specific cis-eQTL genes versus Geuvadis expression. We find that genes with tissue-specific cis-eQTLs are more abundantly expressed in the respective tissues compared with the Geuvadis samples in which we did not observe the cis-eQTLs. [file 13073_2015_152_MOESM10_ESM.pdf]

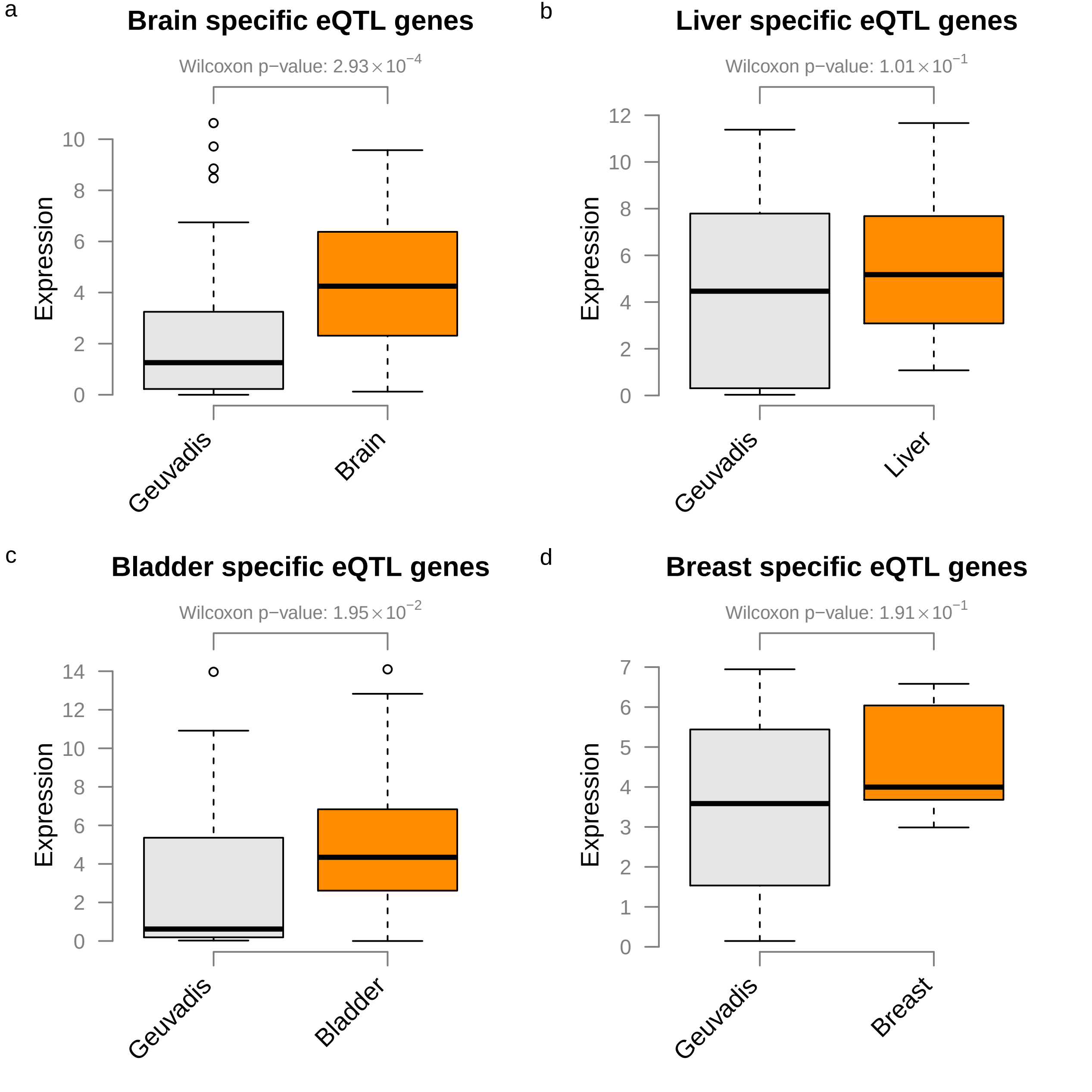
a
Brain specific eQTL genes
Wilcoxon p−value: 2.93×10^{-4}
Expression
0
2
4
6
8
10
Geuvadis
Brain
b
Liver specific eQTL genes
Wilcoxon p−value: 1.01×10^{-1}
Expression
0
2
4
6
8
10
12
Geuvadis
Liver
c
Bladder specific eQTL genes
Wilcoxon p−value: 1.95×10^{-2}
Expression
0
2
4
6
8
10
12
14
Geuvadis
Bladder
d
Breast specific eQTL genes
Wilcoxon p−value: 1.91×10^{-1}
Expression
0
1
2
3
4
5
6
7
Geuvadis
Breast
