# Supplementary material for: Calling genotypes from public RNA-sequencing data enables identification of genetic variants that affect gene-expression levels
Source: Genome Med. 2015 Mar 27;7(1):30. doi: 10.1186/s13073-015-0152-4 (PMC4423486; doi:10.1186/s13073-015-0152-4)
Supplement: Additional file 11: Figure S10. — Expression of example tissue-specific eQTL in different tissues. Here we show three examples of tissue-specific eQTL genes. (a) GSTM5, brain-specific. (b) DDTL, liver-specific. (c) PSCA, bladder-specific. [file 13073_2015_152_MOESM11_ESM.pdf]

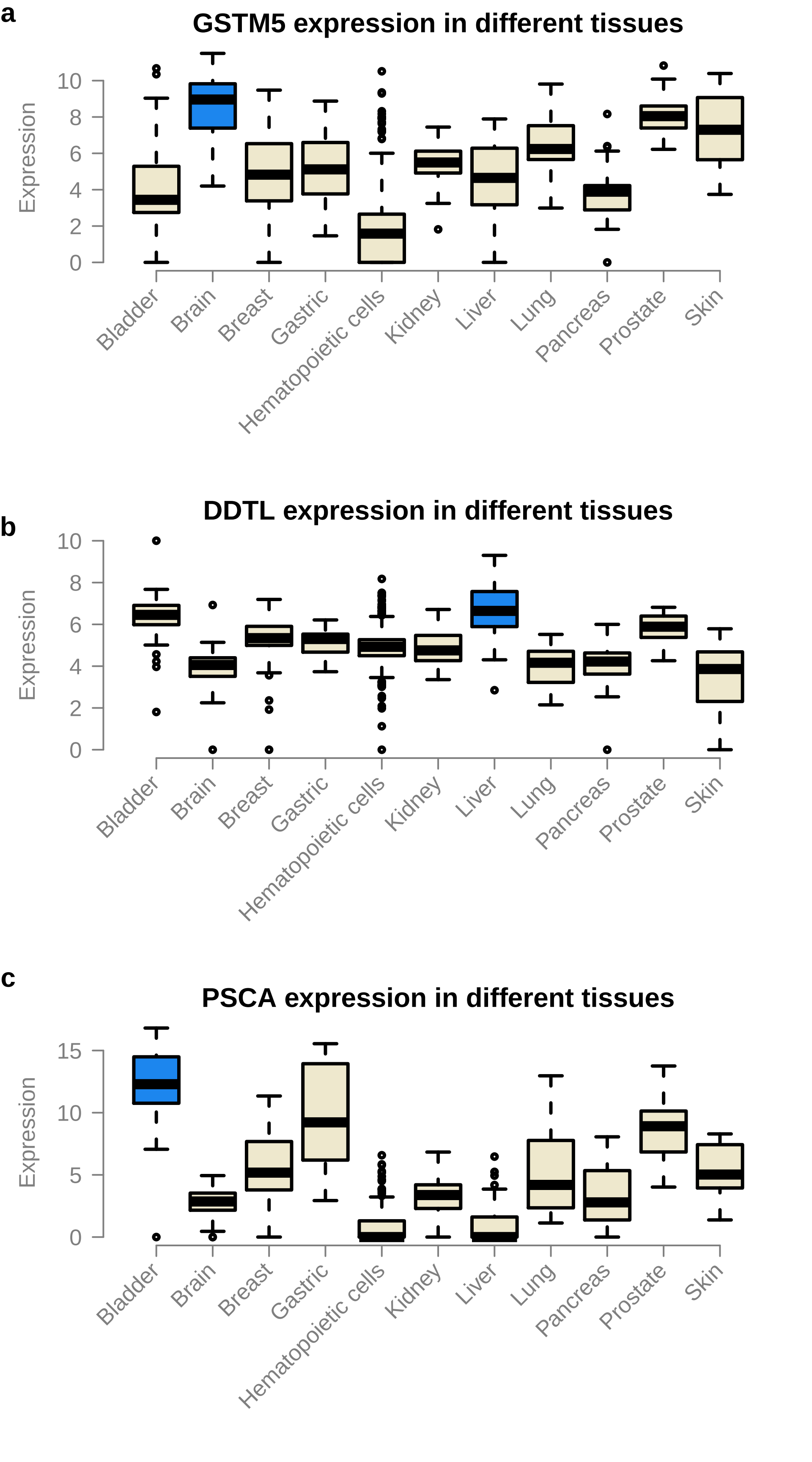
a
GSTM5 expression in different tissues
b
DDTL expression in different tissues
c
PSCA expression in different tissues
Expression
Bladder
Brain
Breast
Gastric
Hematopoietic cells
Kidney
Liver
Lung
Pancreas
Prostate
Skin
