# Supplementary material for: Calling genotypes from public RNA-sequencing data enables identification of genetic variants that affect gene-expression levels
Source: Genome Med. 2015 Mar 27;7(1):30. doi: 10.1186/s13073-015-0152-4 (PMC4423486; doi:10.1186/s13073-015-0152-4)
Supplement: Additional file 12: Figure S11. — Predicted functional impact of ASE variants. The annotation of ASE SNPs predicted impact and effect was performed using SnpEff. (a) Most of the high-impact SNPs have lower expression of the alternative allele. (b) The majority of the SNPs introducing a stop codon have lower expression of the alternative allele. [file 13073_2015_152_MOESM12_ESM.pdf]

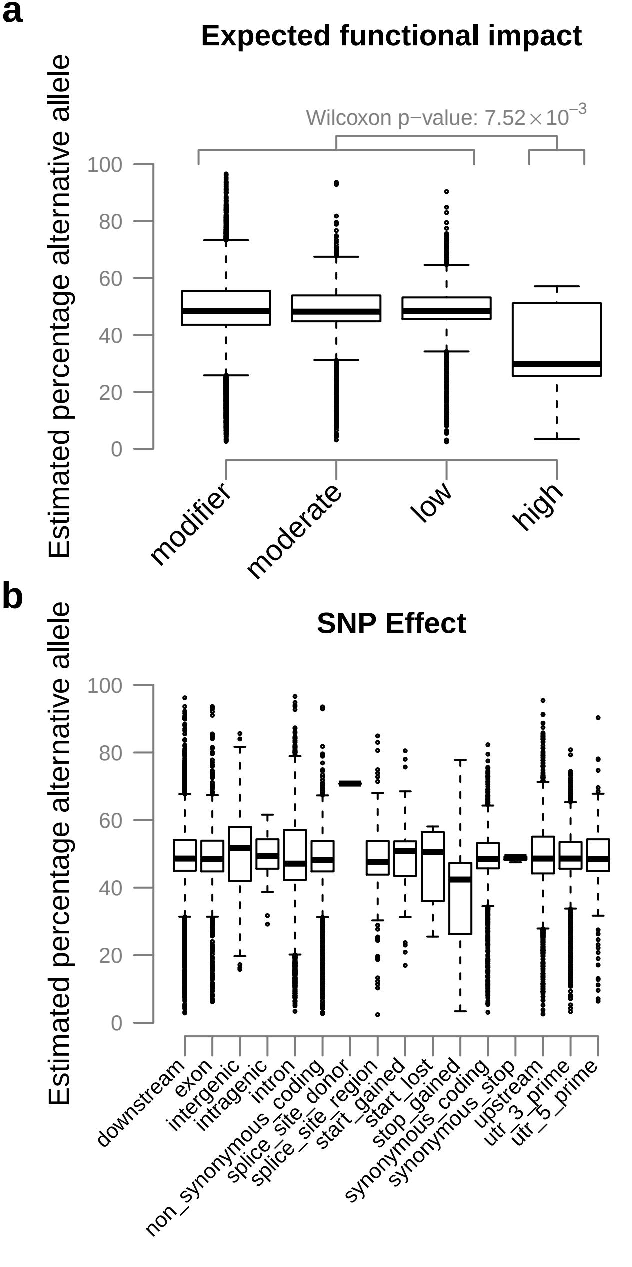
a
Expected functional impact
Wilcoxon p−value: 7.52 × 10^−3
Estimated percentage alternative allele
100
80
60
40
20
0
modifier
moderate
low
high
b
SNP Effect
Estimated percentage alternative allele
100
80
60
40
20
0
downstream
exon
intergenic
intragenic
intron
non_synonymous_coding
splice_site_donor
splice_site_region
start_gained
start_lost
stop_gained
synonymous_coding
synonymous_stop
upstream
utr_3_prime
utr_5_prime
